# Mitochondrially targeted ZFNs for selective degradation of pathogenic mitochondrial genomes bearing large-scale deletions or point mutations

**DOI:** 10.1002/emmm.201303672

**Published:** 2014-02-24

**Authors:** Payam A Gammage, Joanna Rorbach, Anna I Vincent, Edward J Rebar, Michal Minczuk

**Affiliations:** 1 Medical Research Council, Mitochondrial Biology UnitCambridge, UK; 2 Sangamo BioSciences Inc.Richmond, CA, USA

**Keywords:** gene therapy, heteroplasmy, mitochondrial disease, zinc finger nuclease

## Abstract

We designed and engineered mitochondrially targeted obligate heterodimeric zinc finger nucleases (mtZFNs) for site-specific elimination of pathogenic human mitochondrial DNA (mtDNA). We used mtZFNs to target and cleave mtDNA harbouring the m.8993T>G point mutation associated with neuropathy, ataxia, retinitis pigmentosa (NARP) and the “common deletion” (CD), a 4977-bp repeat-flanked deletion associated with adult-onset chronic progressive external ophthalmoplegia and, less frequently, Kearns-Sayre and Pearson's marrow pancreas syndromes. Expression of mtZFNs led to a reduction in mutant mtDNA haplotype load, and subsequent repopulation of wild-type mtDNA restored mitochondrial respiratory function in a CD cybrid cell model. This study constitutes proof-of-principle that, through heteroplasmy manipulation, delivery of site-specific nuclease activity to mitochondria can alleviate a severe biochemical phenotype in primary mitochondrial disease arising from deleted mtDNA species.

## Introduction

Human mitochondria have their own small, circular genome (mtDNA) that encodes essential subunits of the oxidative phosphory-lation (OXPHOS) apparatus. Defects of the mitochondrial genome produce a variety of genetic disorders with diverse clinical manifestations, for which current therapy options are limited to management treatments (Vafai & Mootha, 2012). Population-based studies of primary mtDNA-mediated disease report that average prevalence is 9.2/100,000 (Schaefer *et al*, 2008) and deletions of mtDNA are a common cause of sporadic disease, with single mtDNA deletions accounting for one-third of adults presenting with mitochondrial disease (Chinnery *et al*, 2000).

Delivery and selection of mtDNA in mitochondria in a heritable manner is yet to be achieved, so alternative approaches to genetic therapy of primary mitochondrial diseases are being sought. One of these approaches is based on pathogenic mtDNA mutations being generally heteroplasmic, with observable pathology only present when the ratio of mutated mtDNA exceeds a certain threshold. The selective elimination of mutated mtDNA allows a cell to repopulate with wild-type mtDNA molecules by a yet uncharacterized mechanism of mtDNA copy number maintenance (Carling *et al*, 2011), alleviating the defective mitochondrial function that underlies mtDNA diseases (Taylor *et al*, 1997). Selective degradation of mtDNA bearing point mutations has been achieved by introducing DNA double-strand (ds) breaks using mitochondrially targeted restriction endonucleases (mtRE) in cellular models of disease (Tanaka *et al*, 2002; Alexeyev *et al*, 2008). Also, *in vivo* expression of mtRE efficiently shifted heteroplasmy towards desired mtDNA variants and was reasonably safe in mouse models (Bayona-Bafaluy *et al*, 2005; Bacman *et al*, 2010, 2012). However, there is no appropriate RE for the vast majority of pathological mutations, limiting their usefulness. Furthermore, mtDNA deletions, where a large section of the mitochondrial genome is missing, present a further targeting challenge. Deletions of mtDNA are invariably heteroplasmic and tend to have a lower pathogenicity threshold (∼60%) than point mutations. Typically, the same mtDNA deletion is present in all cells of an affected tissue and many are flanked by short direct repeats. This latter feature renders mtDNA deletions impossible to target with REs as the deletion site will retain one direct repeat, and is therefore indistinguishable from wild-type mtDNA.

Engineered zinc finger nucleases (ZFNs) are chimeric enzymes utilizing the modular Cys_2_His_2_ zinc finger DNA-binding moiety conjugated to the C-terminal catalytic subunit of the type II restriction enzyme *Fok*I (Kim *et al*, 1996; Smith *et al*, 2000). By arranging zinc finger modules appropriately, virtually any DNA sequence can be targeted for nucleolytic cleavage by ZFN. A conventional ZFN strategy consists of targeting two ZFN monomers to bind adjacent sites on complementary DNA strands spanning the target sequence, thus allowing dimerization of the *Fok*I nuclease domain, required to cleave double-stranded DNA. ZFN technology has been used routinely to engineer nuclear genomes in order to add, correct or disrupt genes (Papworth *et al*, 2006; Pearson, 2008) and is currently available commercially and via various protocols for in-house manufacture (Carroll *et al*, 2006; Maeder *et al*, 2008; Kim *et al*, 2011; Sander *et al*, 2011).

In this work, we have expanded the use of mtZFNs through significant design enhancements that permit targeting of pathogenic mitochondrial genomes in cybrid cells. When targeted to pathogenic mtDNA harbouring a point mutation or large-scale deletion, mtZFN expression produces a heteroplasmy shift towards wild-type mtDNA through selective degradation of mutant mtDNA. Moreover, a decrease in the mutant mtDNA load results in phenotypic rescue of the severe bioenergetic dysfunction associated with high levels of partially deleted mtDNA. Thus, we provide a robust method for site-specific editing of mtDNA in patient-derived cybrid cells, with future therapeutic potential.

## Results

### Improved design of mtZFNs

**Figure 1:**
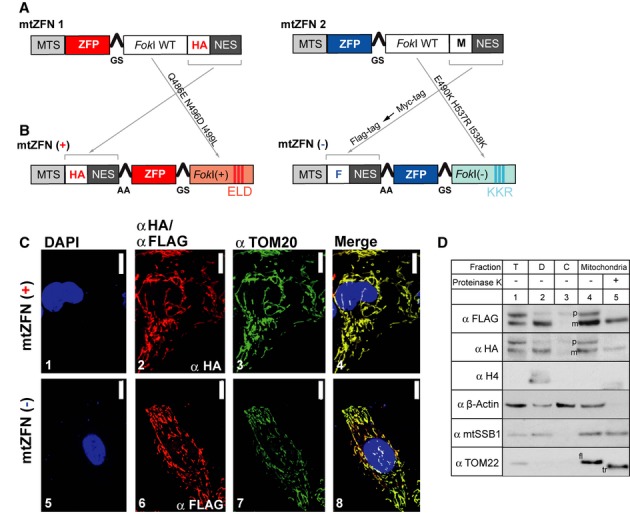
Improved design and localization of mtZFNs.
Schematic structure of previous mtZFN architecture. Mitochondrial targeting is facilitated by a 49-amino acid-long MTS from subunit F1β of human mitochondrial ATP synthase. To ensure that the MTS cleavage site is upstream of the epitope tag, its length was adjusted using proteomic data (Carroll *et al*, 2009) that identified the N-terminus of the mature subunit at Ala48. NES, nuclear export signal; ZFP, zinc finger peptide; M, Myc tag; HA, haemagglutinin tag.Schematic of the novel obligatory heterodimeric mtZFN monomers. “+” and “−”, ELD and KKR modified *Fok*I domain, respectively (Doyon *et al*, 2011); F, FLAG tag; AA, two-alanine linker; GS, glycine-serine linker (Kim *et al*, 1996).The intracellular localization of mtZFNs by immunofluorescence. mtZFN(+) or mtZFN(−) was transiently expressed in HOS 143B cells. Cell nuclei were stained with DAPI (blue, 1 and 5). mtZFN(+) and mtZFN(−) were detected by anti-HA and anti-FLAG antibodies, respectively, and visualized by Alexa Fluor 594-conjugated secondary antibodies (red, 2 and 6). Mitochondria were detected with anti-TOM20/Alexa Fluor 488 antibodies (green, 3 and 7). Co-localization appears yellow on digitally merged images (4 and 8). Scale bars: 10 μm. R8-4 and R13-2 mtZFNs were used in this example (Supplementary Table S2).Location of mtZFNs in subcellular fractions. HOS 143B cells stably transfected with mtZFNs were fractionated into cell debris/nuclei (D, lane 2), cytosol (C, lane 3) and mitochondria (lanes 4–5). The mitochondrial fraction was treated with 25 μg/ml proteinase K (lane 5). “T, total cell lysate. The fractions were analysed by western blotting with anti-HA [mtZFN(+)] and anti-FLAG [mtZFN(−)]. The following marker proteins were used: mtSSB1 (mitochondrial matrix), TOM22 (mitochondrial outer membrane), β-actin (cytosol) and histone H4 (nucleus). “p” indicates mtZFN precursor (still containing MTS), and “m” indicates the mature form. “fl” indicates TOM22 of full length; “tr” indicates proteinase K-truncated TOM22. R8-4 and R13-2 were used in this example. Source data are available for this figure.

Our earlier research found that efficient targeting of ZFNs to human mitochondria requires a mitochondrial targeting signal (MTS) as well as nuclear localization signal (NES) (Minczuk *et al*, 2006). The previously reported architecture of mtZFN monomers (Fig 1A) (Minczuk *et al*, 2008; Minczuk, 2010) was adapted by placing MTS-epitope tag-NES at the N-terminus. In contrast to previous designs, the Myc tag of one of the monomers was replaced with a FLAG tag. We also incorporated modified *Fok*I domains that cleave DNA only when paired as a heterodimer (ELD/KKR). These *Fok*I variants exhibit efficiency comparable to the wild-type architecture, but with a > 40-fold reduction in homodimer activity (Miller *et al*, 2007; Szczepek *et al*, 2007; Doyon *et al*, 2011) (Fig 1B).

In order to experimentally test whether mtZFNs of novel architecture are readily imported into mitochondria, we studied their cellular localization upon transient expression in human osteosarcoma (HOS) 143B cells. Immunofluorescence analysis revealed that mtZFNs co-localize with mitochondria and there is no detectable mtZFN-specific signal from the nucleus (Fig 1C). Further, cell fractionation experiments confirmed the mitochondrial localization of mtZFNs (Fig 1D). This experiment also showed that the MTS is cleaved off from precursor mtZFN proteins, although incompletely; this phenomenon is often observed, even for endogenous mitochondrial proteins expressed at a high level (Maniura-Weber *et al*, 2004). The matured mtZFN form (Fig 1D, “m”) was resistant to proteinase K treatment, consistent with mitochondrial matrix import. In similar conditions, the outer membrane protein TOM22 was truncated by proteinase K (Fig 1D). These results are consistent with mitochondrial localization of this novel obligatory heterodimeric mtZFN architecture in human cells.

### Testing the improved mtZFN design by selective degradation of m.8993T>G NARP point mutation

**Figure 2:**
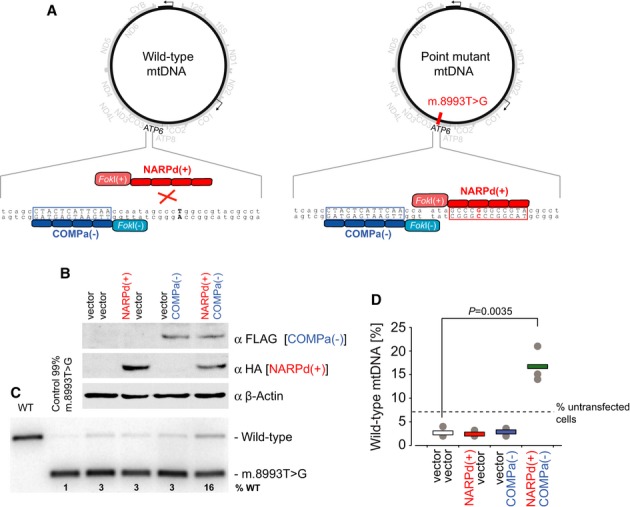
Targeting m.8993T>G with mtZFNs.
Schematic of targeting mtDNA point mutations by mtZFNs. Red box, the binding site for the m.8993T > G-specific mtZFN monomer (NARPd); Blue box, binding site for the companion mtZFN on the opposite strand (COMPa)Western blotting of ˜93% m.8993T > G cybrid cells transfected with NARPd and/or COMPa and vector controls. β-Actin, loading control.RFLP analysis of last cycle hot PCR products (mtDNA nt positions 8339–9334) amplified from total DNA samples of ˜93% m.8993T > G cybrid cells transfected with vectors, NARPd and/or COMPa. Wild-type cells and 99% m.8993T > G cybrids were used as controls.Results of RFLP analysis as per (C) from three independent transfections. *P* value according to two-tailed Student's *t*-test. Source data are available for this figure.

To test the efficacy of our modified mtZFN design, we used previously assembled and optimized zinc finger peptides (ZFPs) with reported specificity to the m.8993T>G substitution (Minczuk *et al*, 2008), which is associated with two mitochondrial diseases: neurogenic muscle weakness, ataxia and retinitis pigmentosa (NARP) and maternally inherited Leigh syndrome (MILS) (Holt *et al*, 1990). According to our strategy (Fig 2A), a mutation-specific mtZFN monomer [Fig 2A, NARPd(+)] and a mtZFN monomer binding to wild-type sequence upstream [Fig 2A, COMPa(−)] would dimerize only at the mutation site and introduce DNA ds breaks, which are inhibitory to mtDNA propagation.

We expressed NARPd(+) and COMPa(−) in cybrid cells harbouring 93% m.8993T>G mtDNA and selected a bulk population of cells expressing both constructs (Fig 2B). Simultaneous expression of NARPd(+) and COMPa(−) for 18 days, as assessed by western blotting, resulted in selective degradation of the m.8993T>G mtDNA molecules, which was accompanied by a shift in heteroplasmy of the cybrids from ∼7% wild-type to ∼17% wild-type, as assessed by RFLP (Fig 2C and D, Supplementary Fig 1). Importantly, NARPd(+)/COMPa(−) rescued heteroplasmy against the natural tendency of the m.8993T>G cybrids cells to select the mutant haplotype over time; in the course of the experiment, mock-transfected cells and cells expressing individual mtZFN monomers demonstrated a heteroplasmy shift from ∼7% to ∼3% of wild-type mtDNA (Fig 2D). Expression of NARPd(+) and/or COMPa(−) did not have any significant effect on mtDNA copy number (Supplementary Fig S2) or cell viability, as informed by co-transfecting equal numbers of cells with mtZFN monomers and fluorescent markers, followed by determining the percentage of marker-positive cells with a flow cytometer (Supplementary Table S1) (Minczuk *et al*, 2008).

Next, we studied the effects of mtZFN expression on mtDNA replication using two-dimensional agarose gel electrophoresis (2D-AGE). One specific issue that this technique can address is whether or not binding of an individual catalytically inactive zinc finger monomer could result in steric hindrance of mtDNA replication, producing stalled replication forks. To address this, we expressed the COMPa(−) mtZFN, which binds wild-type mtDNA sequence, in wild-type HOS 143B cells. The presence of COMPa(−) in the mitochondrial matrix did not produce any appreciable effect on mtDNA replication intermediates in a 2D-AGE analysis (Supplementary Fig 3). This is consistent with mtZFN expression-linked heteroplasmy shifts being attributable to nucleolytic cleavage, rather than replication block.

### Eliminating mtDNA “common deletion” by mtZFNs

**Figure 3:**
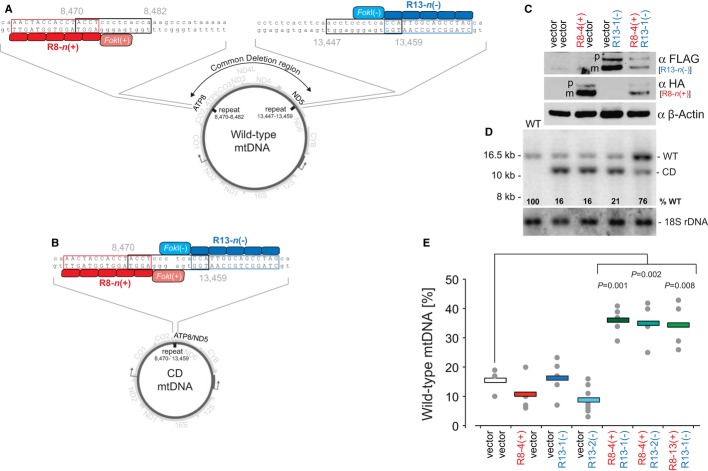
Targeting mtDNA “common deletion” (CD) with mtZFNs.
Wild-type mtDNA is not cleaved as mtZFN R8-*n*(+) and R13-*n*(−) monomers (where *n* is a specific construct number) bind several kilobases apart preventing dimerization of the *Fok*I domain. Red and blue boxes, the binding sites for mtZFN monomers on either side of the CD (Supplementary Table 2); Black boxes, the 13-bp direct repeats.mtDNA harbouring CD is cleaved as adjacent binding of the mtZFN R8-*n*(+) and R13-*n*(−) monomers results in *Fok*I dimerization.Western blotting of bulk populations of H39 CD cybrid cells transfected with the R8-4(+) and/or R13-1(−) constructs and vector controls. β-Actin, loading control.Southern blot analysis of total DNA from wild-type (WT), mock-or mtZFN-transfected cells digested with *Bam*HI and probed with a radioactive mtDNA-specific probe (region: 14986–15607), 18S rDNA probe is used as a loading control.Analysis of mtDNA heteroplasmy in clones expressing CD-specific mtZFNs as indicated. % wild-type mtDNA was measured by Southern blotting. Grey dots indicate individual clones; coloured bars indicate the average value for each mtZFN pair. *P* values calculated in two-tailed Student's *t*-test. Source data are available online for this figure.

Next, we addressed the feasibility of targeting pathogenic large-scale mtDNA deletions in a cybrid cell model. We selected the most frequently occurring large-scale mtDNA deletion of 4977 bp, the so-called common deletion (CD), as the test sequence. This deletion spans a region between two 13-bp direct repeats (nucleotide positions 8470–8482 and 13,447–13,459), containing several transfer RNA and structural genes of the OXPHOS apparatus (Fig 3A). CD often manifests in adult-onset chronic progressive external ophthalmoplegia (CPEO) associated with ptosis and restriction of eye movements and, less frequently, with Kearns-Sayre and Pearson's marrow pancreas syndromes (Grady *et al*, 2013). According to our strategy, wild-type mtDNA is spared as binding sites for the mtZFN monomers are several kilobases apart, preventing dimerization of the nuclease domains (Fig 3A). However, in the CD mtDNA, mtZFN monomers bind adjacently at the CD junction (Fig 3B). This allows dimerization of *Fok*I domains and introduction of DNA ds breaks, which are inhibitory to mtDNA propagation (Fig 3B).

We assembled several ZFN monomers to bind DNA targets downstream of the 8470–8482 repeat (Fig 3, R8-n(+), *n *= construct number) and a further set of ZFNs targeting a site upstream of the 13,447–13,459 repeat (Fig 3, R13-*n*(−), *n *= construct number) (Supplementary Table S2, Supplementary Notes S1, S2). The R8-*n*(+) and R13-*n*(−) design was then tested *in vitro* for specificity to the CD break point site (Supplementary Fig 4). To this end, we synthesized ZFNs in an *in vitro* transcription and translation (IVTT) system. We incubated IVTT-produced R8-*n* and R13-*n* monomers, individually or in pairs, with DNA substrates corresponding to the joining site resulting from CD, or with DNA probes containing mtDNA wild-type sequence proximal to the 8470–8482 or 13,447–13,459 direct repeats (Supplementary Note 3) (Minczuk *et al*, 2010). This analysis identified several R8-*n*(+)/R13-*n*(−) pairs that cleaved the CD-specific DNA, while sparing the wild-type substrates; these were selected for further analysis (Supplementary Fig S4).

Next, we tested whether these selected mtZFNs were effective in degrading CD mtDNA in heteroplasmic cells (Porteous *et al*, 1998). To determine this, we transfected the R8-4(+) and R13-1(−) mtZFN variants individually or concurrently into HOS H39 cells (Porteous *et al*, 1998), which harbour on average ∼85% CD mtDNA, and selected a bulk population of cells expressing mtZFNs (Fig 3C). We analysed mtDNA heteroplasmy in transfected H39 cells using Southern blotting, allowing the detection of expected mtDNA haplotypes, as well as potential, undesired recombined mtDNA species (Bacman *et al*, 2009) (Fig 3D). This analysis showed that simultaneous expression of both the R8-4(+) and R13-1(−) mtZFNs for 18 days led to efficient degradation of CD mtDNA, shifting the proportion of wild-type mtDNA from ∼15% to ∼76% (Fig 3D). Mock-transfected cells or cells expressing high levels of either R8-4(+) or R13-1(−) individually did not show any appreciable changes in heteroplasmy levels and no recombined mtDNA species were detected in any conditions (Fig 3D). The presence of R8-4(+) and/or R13-1(−) monomers did not have any significant effect on mtDNA copy number (Supplementary Fig S5). These results demonstrate that CD-specific mtZFNs are capable of reducing pathogenic mtDNA deletion content in a cybrid cell model.

In order to provide further evidence for selective degradation of pathogenic rearranged mtDNA by mtZFNs, we retested the R8-4(+)/R13-1(−) combination and two other CD-specific mtZFN pairs (Supplementary Fig S5) in clonal H39 cell lines transfected with appropriate DNA constructs. There were two key reasons for testing the mtZFNs in clonal cell lines. First, we observed that the heteroplasmy shift leading to dominance of wild-type mtDNA was accompanied by a considerable growth advantage (Supplementary Fig S6), so it was conceivable that the bulk population will over-represent cells with higher levels of wild-type mtDNA rather than demonstrate the true relationship between wild-type and CD mtDNA in individual cells. Secondly, homogenous populations of cells were required for an extracellular flux analysis of OXPHOS function (see below). In this experiment, HOS H39 cells were transfected with the following combination of mtZFN constructs: R8-4(+)/R13-1(−), R8-4(+)/R13-2(−) and R8-13(+)/R13-1(−). Additionally, R8-4(+), R13-1(−) and R13-2(−) were tested individually. When representative clones were analysed to assess wild-type/CD mtDNA ratio by Southern blotting, all mtZFN pairs tested were capable of eliminating the pathogenic mtDNA deletion (Fig 3E, Supplementary Fig S5). However, on average, they exhibited lower levels of wild-type mtDNA as compared with the initial bulk population. Of note, a clone that demonstrated ∼70% wild-type mtDNA was also observed in this experiment; however, it was removed from Fig 3E after statistical analysis as it lay >10 standard deviations from the mean.

### Rescue of mtDNA “common deletion”-associated mitochondrial defect by mtZFNs

**Figure 4:**
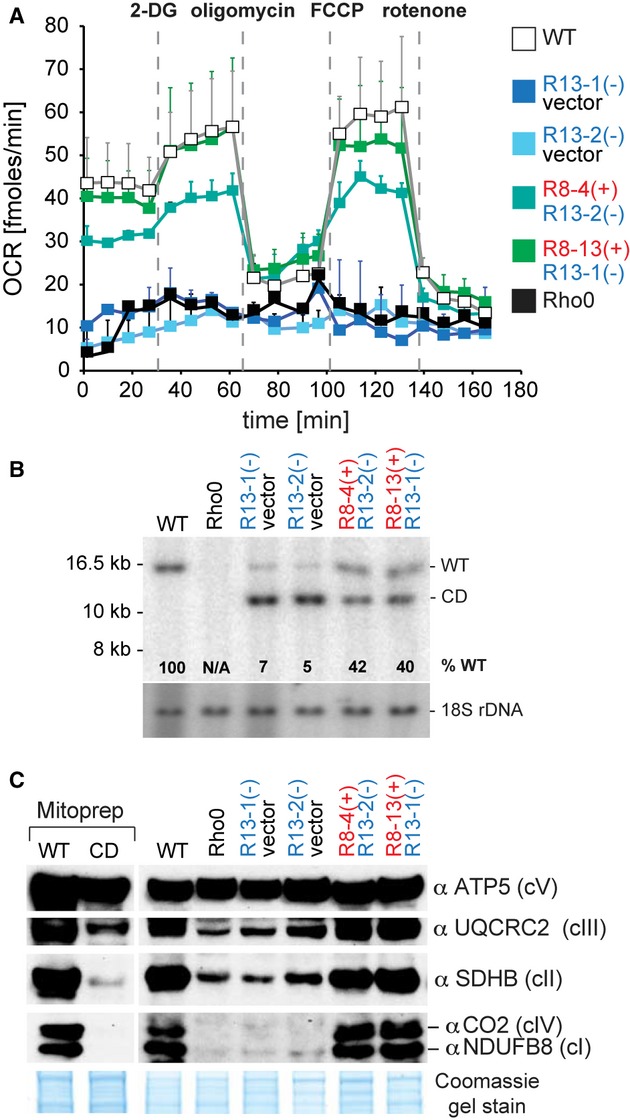
Recovery of OXPHOS function upon elimination of CD mtDNA by mtZFNs.
Oxygen consumption rate (OCR) measured in quadruplicate for wild-type (WT, white), Rho0 (black) and clonal cells expressing CD-specific mtZFNs from the same transfection/selection experiment.Southern blot analysis of total DNA from wild-type (WT), Rho0 and mock-or mtZFN-transfected clonal cells used in OCR analysis.Western blot analysis of steady-state levels of OXPHOS subunits in clonal cell lines as per (A) and (B). Mitochondrial preparations of WT and CD cells (Mitoprep) were also analysed in this experiment. Coomassie gel staining was used as a control for protein loading. Source data are available for this figure.

Next, we set out to determine whether mtZFN-dependent shifts in heteroplasmy from CD to wild-type mtDNA were associated with rescue of mitochondrial respiratory function. The region of CD spans seven genes coding for structural subunits of complexes I, IV and V and also several transfer RNA (Fig 3A). Consequently, high levels of CD mtDNA are associated with a generalized OXPHOS defect. We measured oxygen consumption rate (OCR) in controls and clonal H39 cells expressing CD-specific mtZFNs in an extracellular flux analysis. OCR of control H39 cells expressing single mtZFN monomers [R13-1(−) or R13-2(−)] with high levels of CD was virtually indistinguishable from that of mtDNA-less (Rho0) cells (Fig 4A, blue and black traces, respectively), consistent with data published previously (Porteous *et al*, 1998). The specific shift in mtDNA heteroplasmy from CD towards wild-type produced by expression of R8-4(+)/R13-2(−) or R8-13(+)/R13-1(−) mtZFNs (Fig 4B) resulted in rescue of OCR (Fig 4A, green traces). Furthermore, degradation of CD mtDNA in clonal H39 HOS cell lines expressing mtZFN pairs resulted in increased abundance of respiratory complex subunits as compared to control H39 or Rho0 cells **(**Fig 4C**)**. These data confirm that the defect of mitochondrial gene expression and OXPHOS function in H39 HOS cells displaying high levels of CD mtDNA is corrected by mtZFNs specifically degrading CD genomes, allowing wild-type mtDNA to repopulate the cell.

## Discussion

This work is concerned with solving the longstanding problem of how to manipulate the human mitochondrial genome and, eventually, correct point mutations and deletions in mtDNA that are associated with human pathologies. Currently, these pathologies are untreatable and difficult for clinicians to manage; frequently, they lead to severe dysfunction in early childhood and premature death; thus, finding a solution is a major priority (Pfeffer *et al*, 2013). Here, we describe a significant step towards achieving this goal using engineered zinc finger nucleases selected to target and cleave predetermined loci in mtDNA.

We report a redesigned, conventional dimeric mtZFN architecture to provide a safe and universal approach for genetic manipulation of the mitochondrial genome. Our previous designs for mtZFN monomers were inefficient when targeting mtDNA point mutations in living cells (Minczuk *et al*, 2008), despite exhibiting high specificity *in vitro* (Minczuk, 2010). This prompted us to target a specific point mutant sequence in human mtDNA with a single-chain ZFN that carries two cleavage domains linked to the same protein (Minczuk *et al*, 2008). The strategy of linking two cleavage domains, although successful in targeting mtDNA point mutations, cannot be used to target the break points of many mtDNA deletions (see Introduction). Another limitation of single-chain ZFNs is that the enzyme may be constitutively active as a nuclease, partially compromising its safe use. An attractive feature of the more conventional dimeric ZFN scheme is that the cleavage reagent is assembled at the target and is otherwise inactive. This encouraged us to revisit the initial approach and improve the dimeric mtZFN architecture.

Our earlier designs for mtZFN monomers, in addition to the N-terminal MTS, contained epitope tags and the NES sequence at the C-terminus following the wild-type *Fok*I domain (Minczuk, 2010; Minczuk *et al*, 2010) (Fig 1A). The incorporation of NES, in addition to the MTS, has been shown to be necessary to circumvent the issue of undesired nuclear localization of mtZFN and other proteins (Minczuk *et al*, 2006; Suzuki *et al*, 2010). Other studies have shown that this was likely attributable to an internal nuclear localization signal (NLS) that overlaps residues important for contacting DNA in zinc finger proteins (Matheny *et al*, 1994; Fernandez-Martinez *et al*, 2003). The probable key weaknesses of the previous mtZFN architecture, limiting its safety and/or efficacy, were: (i) potential interference of extra domains (HA/Myc and NES) with *Fok*I at the C-terminus, (ii) destabilization of mtZFNs by the Myc tag, as observed for other mitochondrially targeted proteins studied in our laboratory, (iii) tendency of wild-type *Fok*I to form cleavage-competent homodimers, resulting in off-target cleavage events and subsequent depletion of mtDNA copy number in conditions where only one mtZFN monomer is present in mitochondria (Minczuk *et al*, 2008).

Having addressed these issues (Fig 1b), our novel obligatory heterodimeric mtZFN architecture has been demonstrated to localize to mitochondria (Fig 1C and D) and selectively eliminate pathogenic mtDNA bearing the m.8993T>G point mutation (Fig 2). Importantly, this effect was shown not to be mediated by mtDNA replication stalling and subsequent replicative advantage (Supplementary Fig S3). Compared to the previously reported single-chain ZFN targeting m.8993T>G, our current design is superior in shifting heteroplasmy while using the same, mutant-specific DNA-binding zinc finger peptide (NARPd). Long-term expression of the m.8993T>G-specific single-chain ZFN resulted in ∼twofold increase in wild-type mtDNA content as compared to the mock control (Minczuk *et al*, 2008). The dimeric mtZFN targeting the same mutation in this study produced ∼fivefold more wild-type mtDNA relative to the controls (Fig 2). Importantly, the improved mtZFN monomers did not exert any effect on mtDNA when expressed individually (Fig 2).

In order to expand the use of mtZFNs for mtDNA heteroplasmy manipulation, beyond pathogenic point mutations, we targeted the most frequently occurring disease-associated large-scale mtDNA deletion, the “common deletion” (CD) (Fig 3). Expression of several combinations of CD-specific mtZFNs led to a reduction in mutant mtDNA haplotype load below the pathogenicity threshold. Subsequent repopulation of wild-type mtDNA produced a profound recovery of OXPHOS function in treated cells (Fig 4). With several combinations of mtZFNs capable of specifically degrading targeted mtDNA variants, these data predict that the mtZFN approach could be advantageous to patients with primary mitochondrial diseases and may constitute a feasible genetic therapy approach in the future.

The highly efficient shift in heteroplasmy from mutant to wild-type for the CD cell line contrasted with the shift observed in the m.8993T>G cells. There are two plausible reasons for this: (i) there is a far lower starting quantity of wild-type mtDNA in the m.8993T>G cell line; therefore, the rapid, initial elimination of mutant mtDNA leaves very little capacity for mtDNA repopulation and (ii) the m.8993T>G cell line has a tendency to shift heteroplasmy towards the mutant haplotype (Fig 2D), both masking the efficacy of mtZFNs and exacerbating the previously stated effect. Further research is in progress in our laboratory to verify the hypothesis that a higher initial load of wild-type mtDNA would result in more efficient heteroplasmy shifts of m.8993T>G by mtZFNs. If this assumption is correct, several sequential transfections could produce complete (or near complete) elimination of mutant mtDNA.

Very recently, an alternative method for specific elimination of mutant mitochondrial genomes was reported, utilizing mitochondrially targeted TAL-effector nucleases (mitoTALENs) (Bacman *et al*, 2013). Similar to our experience with mtZFNs, expression of mitoTALENs led to reductions in deletion and point mutant mtDNA. Bacman *et al* also targeted CD; however, a different point mutation (m.14459G>A) was eliminated in their study. At this stage in the development of both methodologies, it is not clear which, if either, solution provides an answer to the current problem of therapeutic manipulation of mitochondrial heteroplasmy. With advancements in the field of engineered nuclease technology aimed at minimizing the risk of use *in vivo*, or even moving into early-phase clinical trials (Palpant & Dudzinski, 2013), our work and the results obtained for mitoTALENs should encourage further experiments in animal models to ascertain safe use and efficacy. However, prior to this, several important issues and challenges must be addressed, namely understanding the relative specificity of each designer nuclease platform and harnessing efficient methods for delivering these nucleases *in vivo* (Gaj *et al*, 2013). The considerable size of TALEN assemblies may also become particularly important in the context of construct packaging into size-restricted vectors such as the recombinant adeno-associated virus (AAV), which has already been shown to accommodate and deliver ZFN constructs (Ellis *et al*, 2013). This issue might suggest that the development of new systems for mitoTALEN delivery will be a critical hurdle to leap in future research.

Our success in delivering ZFNs to mitochondria, altering the ratio of desired haplotypes also points the way to future applications for genetic manipulation of mtDNA in basic research. Achieving this end is of great importance to the entire mitochondrial research community, as we are currently unable to deliver exogenous nucleic acids to mitochondria, obstructing all approaches towards engineering mammalian mtDNA in living cells (Lightowlers, 2011).

## Materials and Methods

### Transfection and cell culture

Wild-type HOS 143B cells and cybrid HOS 143B cells (Porteous *et al*, 1998) were maintained in DMEM supplemented with 4.5 g/L D-glucose, 2 mM l-glutamine, 1 mM sodium pyruvate and 10% FCS in standard human cell culture conditions. Cultures of CD cybrid cells were additionally supplemented with 50 μg/ml uridine. Plasmids containing either NARPd(+), COMPa(−) R8-*n*(+) or R13-*n*(−) and empty vectors were linearized and delivered to cells in culture using Lipofectamine 2000 (Invitrogen) according to the manufacturer's instructions. Cells expressing stably integrated vectors and transgenes were sequentially selected by supplementing standard culture media with 1 mg/ml neomycin and 4 μg/ml blasticidin for 18 days each.

### Cell fractionation

Cell fractionation was performed as described in (Minczuk *et al*, 2011) without the sucrose gradient step.

### Immunodetection of proteins

The localization of proteins by immunofluorescence in fixed HOS 143B cells was performed as described previously (Minczuk, 2010). The following antibodies were used: rabbit anti-TOM20 (Santa Cruz), Alexa Fluor 488 anti-rabbit mouse anti-FLAG (Sigma, F1804, 1:200), Alexa Fluor 594 anti-mouse (Life Technologies, A11006, 1:200), rat anti-HA (Roche, 11867431001, 1:200), Alexa Fluor 594 anti-rat (Life Technologies, A11001, 1:200). Immunofluorescence images were captured using a Nikon N-SIM confocal microscope.

Western blotting analysis was performed as previously (Minczuk *et al*, 2011), and membranes were probed with the following primary antibodies: anti-FLAG and anti-HA as above, mouse anti-OXPHOS cocktail (Mitosciences, MS601, 1:300), mouse anti-TOM22 (Abcam, ab10436, 1:5000), mouse anti-β-actin (Sigma, A2228, 1:100,000), rabbit anti-SSB1 (kindly donated by Prof. D. Kang, 1:4000), rabbit anti-histone H4 (Abcam, ab10158; 1:5000). Secondary antibodies used were HRP-conjugated goat antibodies to rabbit (Promega, W401B; 1:2000), mouse (Promega, W402B, 1:2000) and rat (Santa Cruz, SC2065, 1:1000).

### Last cycle hot RFLP

Total cellular DNA was isolated using a DNeasy Blood and Tissue kit (Qiagen) according to the manufacturer's instructions. Approximately 200 ng DNA was used as template for a 15-cycle cold PCR amplification using KOD polymerase (TaKaRa). A final cycle of PCR amplification was then performed following the addition of 2 mM ^32^P-^α^dCTP and the resulting products were purified using a PCR Purification kit (Qiagen) according to the manufacturer's instructions. Purified PCR products were then digested with 20 U *Sma*I according to the manufacturer's instructions and resolved on 1% agarose gel at 80 V for 1 h. Gels were dried onto DE81 paper (Whatman) and exposed to a storage phosphor screen for 12 h before imaging with a Typhoon 9410 scanner and processing with ImageQuant software (GE Healthcare). Primer sequences were as follows:

mt. 8339-9334:    5′ CCACCCAACAATGACTAATC 3′

5′ GTATGAGGAGCGTTATGGAG 3′

### Southern blotting

Total cellular DNA was isolated using a DNeasy Blood and Tissue kit (Qiagen) according to the manufacturer's instructions. Approximately 2.5 μg DNA was digested with 20 U *Bam*HI (New England Biolabs), and digest fragments were separated on 0.6% agarose gel for approximately 20 h at room temperature. Gels were blotted onto nylon membranes (GE Healthcare) and probed with a ^32^P-radiolabelled probes in hybridization buffer (7% SDS, 0.5 M sodium phosphate buffer, pH 7.4) for 18 h at 65°C. Membranes were washed three times in 1× SSC with 0.1% SDS and exposed to a storage phosphor screen for 24–96 h before imaging with a Typhoon 9410 scanner and processing with ImageQuant software (GE Healthcare). Probe primer sequences were as follows:

mt. 14986-15607:    5′ CTACCTTCACGCCAATGG 3′

5′ CGATCCGTCCCTAACAAA 3′

mt. 10202-10806:    5′ CGTCCCTTTCTCCATAAAATTC 3′

5′ TTGGAAAGTCATGTCAGTGGTAG 3′

18S rDNA: 5′ GTTGGTGGAGCGATTTGTCT 3′

5′ GGCCTCACTAAACCATCCAA 3′

**The paper explained**

**Problem**

Mitochondria contain hundreds of copies of a small, circular genome per cell. Mutations and rearrangements of mitochondrial DNA (mtDNA) are a common cause of human disease. Pathogenic mtDNA variants often co-exist with wild-type mtDNA in a single cell, a phenomenon termed heteroplasmy. A mutation-specific threshold in the ratio of mutant to wild-type mtDNA must be exceeded for a phenotype to manifest. Currently, these pathologies are untreatable and difficult for clinicians to manage; frequently, they lead to severe dysfunction in early childhood and premature death; thus, finding a solution is a major priority.

**Results**

Here, we report on mitochondrially targeted engineered zinc finger nucleases (mtZFN), chimeric DNA-cleaving enzymes with universal directed DNA sequence specificity. When targeted to pathogenic mtDNA, mtZFN expression achieves phenotypic rescue of a severe mtDNA-mediated dysfunction by shifting heteroplasmy towards wild-type through selective degradation of mutant mtDNA.

**Impact**

We provide a robust method for site-specific editing of mtDNA in patient-derived cells with future therapeutic potential.

### Extracellular flux analysis

Extracellular flux analysis of OXPHOS function was performed as described previously (Rorbach *et al*, 2012), with the details provided in the Supplementary Materials and Methods.

### Other methods

Design and *in vitro* testing of ZFPs, generation of mtZFN and two-dimensional agarose gel electrophoresis are presented in the Supplementary Materials and Methods.

## References

[b1] Alexeyev MF, Venediktova N, Pastukh V, Shokolenko I, Bonilla G, Wilson GL (2008). Selective elimination of mutant mitochondrial genomes as therapeutic strategy for the treatment of NARP and MILS syndromes. Gene Ther.

[b2] Bacman SR, Williams SL, Duan D, Moraes CT (2012). Manipulation of mtDNA heteroplasmy in all striated muscles of newborn mice by AAV9-mediated delivery of a mitochondria-targeted restriction endonuclease. Gene Ther.

[b3] Bacman SR, Williams SL, Garcia S, Moraes CT (2010). Organ-specific shifts in mtDNA heteroplasmy following systemic delivery of a mitochondria-targeted restriction endonuclease. Gene Ther.

[b4] Bacman SR, Williams SL, Moraes CT (2009). Intra-and inter-molecular recombination of mitochondrial DNA after in vivo induction of multiple double-strand breaks. Nucleic Acids Res.

[b5] Bacman SR, Williams SL, Pinto M, Peralta S, Moraes CT (2013). Specific elimination of mutant mitochondrial genomes in patient-derived cells by mitoTALENs. Nat Med.

[b6] Bayona-Bafaluy MP, Blits B, Battersby BJ, Shoubridge EA, Moraes CT (2005). Rapid directional shift of mitochondrial DNA heteroplasmy in animal tissues by a mitochondrially targeted restriction endonuclease. Proc Natl Acad Sci USA.

[b7] Carling PJ, Cree LM, Chinnery PF (2011). The implications of mitochondrial DNA copy number regulation during embryogenesis. Mitochondrion.

[b8] Carroll D, Morton JJ, Beumer KJ, Segal DJ (2006). Design, construction and in vitro testing of zinc finger nucleases. Nat Protoc.

[b9] Carroll J, Fearnley IM, Wang Q, Walker JE (2009). Measurement of the molecular masses of hydrophilic and hydrophobic subunits of ATP synthase and complex I in a single experiment. Anal Biochem.

[b10] Chinnery PF, Johnson MA, Wardell TM, Singh-Kler R, Hayes C, Brown DT, Taylor RW, Bindoff LA, Turnbull DM (2000). The epidemiology of pathogenic mitochondrial DNA mutations. Ann Neurol.

[b11] Doyon Y, Vo TD, Mendel MC, Greenberg SG, Wang J, Xia DF, Miller JC, Urnov FD, Gregory PD, Holmes MC (2011). Enhancing zinc-finger-nuclease activity with improved obligate heterodimeric architectures. Nat Methods.

[b12] Ellis BL, Hirsch ML, Porter SN, Samulski RJ, Porteus MH (2013). Zinc-finger nuclease-mediated gene correction using single AAV vector transduction and enhancement by Food and Drug Administration-approved drugs. Gene Ther.

[b13] Fernandez-Martinez J, Brown CV, Diez E, Tilburn J, Arst HN, Penalva MA, Espeso EA (2003). Overlap of nuclear localisation signal and specific DNA-binding residues within the zinc finger domain of PacC. J Mol Biol.

[b14] Gaj T, Gersbach CA, Barbas CF (2013). ZFN, TALEN, and CRISPR/Cas-based methods for genome engineering. Trends Biotechnol.

[b15] Grady JP, Campbell G, Ratnaike T, Blakely EL, Falkous G, Nesbitt V, Schaefer AM, McNally RJ, Gorman GS, Taylor RW Disease progression in patients with single, large-scale mitochondrial DNA deletions. Brain.

[b16] Holt IJ, Harding AE, Petty RK, Morgan-Hughes JA (1990). A new mitochondrial disease associated with mitochondrial DNA heteroplasmy. Am J Hum Genet.

[b17] Kim S, Lee MJ, Kim H, Kang M, Kim JS (2011). Preassembled zinc-finger arrays for rapid construction of ZFNs. Nat Methods.

[b18] Kim YG, Cha J, Chandrasegaran S (1996). Hybrid restriction enzymes: zinc finger fusions to Fok I cleavage domain. Proc Natl Acad Sci USA.

[b19] Lightowlers RN (2011). Mitochondrial transformation: time for concerted action. EMBO Rep.

[b20] Maeder ML, Thibodeau-Beganny S, Osiak A, Wright DA, Anthony RM, Eichtinger M, Jiang T, Foley JE, Winfrey RJ, Townsend JA (2008). Rapid “open-source” engineering of customized zinc-finger nucleases for highly efficient gene modification. Mol Cell.

[b21] Maniura-Weber K, Goffart S, Garstka HL, Montoya J, Wiesner RJ (2004). Transient overexpression of mitochondrial transcription factor A (TFAM) is sufficient to stimulate mitochondrial DNA transcription, but not sufficient to increase mtDNA copy number in cultured cells. Nucleic Acids Res.

[b22] Matheny C, Day ML, Milbrandt J (1994). The nuclear localization signal of NGFI-A is located within the zinc finger DNA binding domain. J Biol Chem.

[b23] Miller JC, Holmes MC, Wang J, Guschin DY, Lee YL, Rupniewski I, Beausejour CM, Waite AJ, Wang NS, Kim KA (2007). An improved zinc-finger nuclease architecture for highly specific genome editing. Nat Biotechnol.

[b24] Minczuk M (2010). Engineered zinc finger proteins for manipulation of the human mitochondrial genome. Methods Mol Biol.

[b25] Minczuk M, He J, Duch AM, Ettema TJ, Chlebowski A, Dzionek K, Nijtmans LG, Huynen MA, Holt IJ (2011). TEFM (c17orf42) is necessary for transcription of human mtDNA. Nucleic Acids Res.

[b26] Minczuk M, Kolasinska-Zwierz P, Murphy MP, Papworth MA (2010). Construction and testing of engineered zinc-finger proteins for sequence-specific modification of mtDNA. Nat Protoc.

[b27] Minczuk M, Papworth MA, Kolasinska P, Murphy MP, Klug A (2006). Sequence-specific modification of mitochondrial DNA using a chimeric zinc finger methylase. Proc Natl Acad Sci USA.

[b28] Minczuk M, Papworth MA, Miller JC, Murphy MP, Klug A (2008). Development of a single-chain, quasi-dimeric zinc-finger nuclease for the selective degradation of mutated human mitochondrial DNA. Nucleic Acids Res.

[b29] Palpant NJ, Dudzinski D (2013). Zinc finger nucleases: looking toward translation. Gene Ther.

[b30] Papworth M, Kolasinska P, Minczuk M (2006). Designer zinc-finger proteins and their applications. Gene.

[b31] Pearson H (2008). Protein engineering: the fate of fingers. Nature.

[b32] Pfeffer G, Horvath R, Klopstock T, Mootha VK, Suomalainen A, Koene S, Hirano M, Zeviani M, Bindoff LA, Yu-Wai-Man P (2013). New treatments for mitochondrial disease-no time to drop our standards. Nat Rev Neurol.

[b33] Porteous WK, James AM, Sheard PW, Porteous CM, Packer MA, Hyslop SJ, Melton JV, Pang CY, Wei YH, Murphy MP (1998). Bioenergetic consequences of accumulating the common 4977-bp mitochondrial DNA deletion. Eur J Biochem.

[b34] Rorbach J, Gammage PA, Minczuk M (2012). C7orf30 is necessary for biogenesis of the large subunit of the mitochondrial ribosome. Nucleic Acids Res.

[b35] Sander JD, Dahlborg EJ, Goodwin MJ, Cade L, Zhang F, Cifuentes D, Curtin SJ, Blackburn JS, Thibodeau-Beganny S, Qi Y (2011). Selection-free zinc-finger-nuclease engineering by context-dependent assembly (CoDA). Nat Methods.

[b36] Schaefer AM, McFarland R, Blakely EL, He L, Whittaker RG, Taylor RW, Chinnery PF, Turnbull DM (2008). Prevalence of mitochondrial DNA disease in adults. Ann Neurol.

[b37] Smith J, Bibikova M, Whitby FG, Reddy AR, Chandrasegaran S, Carroll D (2000). Requirements for double-strand cleavage by chimeric restriction enzymes with zinc finger DNA-recognition domains. Nucleic Acids Res.

[b38] Suzuki Y, Holmes JB, Cerritelli SM, Sakhuja K, Minczuk M, Holt IJ, Crouch RJ (2010). An upstream open reading frame and the context of the two AUG codons affect the abundance of mitochondrial and nuclear RNase H1. Mol Cell Biol.

[b39] Szczepek M, Brondani V, Buchel J, Serrano L, Segal DJ, Cathomen T (2007). Structure-based redesign of the dimerization interface reduces the toxicity of zinc-finger nucleases. Nat Biotechnol.

[b40] Tanaka M, Borgeld HJ, Zhang J, Muramatsu S, Gong JS, Yoneda M, Maruyama W, Naoi M, Ibi T, Sahashi K (2002). Gene therapy for mitochondrial disease by delivering restriction endonuclease SmaI into mitochondria. J Biomed Sci.

[b41] Taylor RW, Chinnery PF, Turnbull DM, Lightowlers RN (1997). Selective inhibition of mutant human mitochondrial DNA replication in vitro by peptide nucleic acids. Nat Genet.

[b42] Vafai SB, Mootha VK (2012). Mitochondrial disorders as windows into an ancient organelle. Nature.

