# Supplementary material for: Mitochondrially targeted ZFNs for selective degradation of pathogenic mitochondrial genomes bearing large-scale deletions or point mutations
Source: EMBO Mol Med. 2014 Feb 24;6(4):458–66. doi: 10.1002/emmm.201303672 (PMC3992073; doi:10.1002/emmm.201303672)
Supplement: Supplementary file 1 [file emmm0006-0458-sd1.pdf]

EMBO Molecular Medicine

Mitochondrially-targeted ZFNs for selective degradation of pathogenic mitochondrial genomes bearing large-scale deletions or point mutations

Payam A. Gammage, Joanna Rorbach, Anna I. Vincent, Edward J. Rebar & Michal Minczuk

Corresponding author: Michal Minczuk, Medical Research Council

---

Review timeline:

Submission date:	14 November 2013
Editorial Decision:	09 December 2013
Revision received:	06 January 2014
Editorial Decision:	08 January 2014
Revision received:	09 January 2014
Accepted:	13 January 2014

Transaction Report:

(Note: With the exception of the correction of typographical or spelling errors that could be a source of ambiguity, letters and reports are not edited. The original formatting of letters and referee reports may not be reflected in this compilation.)

Editor: Céline Carret

1st Editorial Decision 09 December 2013

Thank you for the submission of your manuscript to EMBO Molecular Medicine. We have now heard back from the three referees whom we asked to evaluate your manuscript.

As you will see from the comments below, the three referees are rather supportive of the study but still require additional explanations and discussions (all three referees), to confirm the main results using other technologies (referee 2), and to provide better imaging (referee 3).

We would welcome the submission of a revised version for further consideration. However, we would like to suggest shortening the study as it is succinct as referee 2 puts it, and we feel that a report-format would be better appropriate. As we have a maximum of 3 (sometimes 4) figures, we would suggest to either reorganise the figures or move some data to supplemental information.

Please note that it is EMBO Molecular Medicine policy to allow a single round of revision in order to avoid the delayed publication of research findings. Consequently, acceptance or rejection of the manuscript will depend on the completeness of your responses included in the next version of the manuscript.

EMBO Molecular Medicine has a "scooping protection" policy, whereby similar findings that are published by others during review or revision are not a criterion for rejection. Should you decide to submit a revised version, I do ask that you get in touch after three months if you have not completed it, to update us on the status.

Please also contact us as soon as possible if similar work is published elsewhere. If other work is published we may not be able to extend the revision period beyond three months.

I look forward to seeing a revised form of your manuscript as soon as possible.

***** Reviewer's comments *****

Referee #1 (Remarks):

This paper describes a significant improvement in the ZFN approach to selectively degrading mutated mtDNA. I have relatively few comments:

1. One of my main concerns is the relative inefficiency of the ZFN approach to change the level of heteroplasmy in the m.8993T>C cells - there is a relatively small shift in heteroplasmy and much less than in the single deletion cell lines. Is there an explanation for this and do the authors feel this will reduce the utility of this approach.

2. As far as I can tell the authors do not specifically say how long they left the cells after transfection/selection - just wondered if they had varied this to see if they get better loss of mutated mtDNA. Is it possible to completely remove the mutated form?

3. Figure 4c is somewhat confusing - the WT there are three bands but only two proteins. Also the ratio of CO2 to NDUFB8 is very different from the cells treated with the ZFNs

Referee #2 (Comments on Novelty/Model System):

Appropriate cellular models used in proof of principle experiments

Referee #2 (Remarks):

The paper by Gammage et al investigates the use of mitochondrially-targeted zinc-finger nucleases (mtZFNs) to human cells harbouring pathogenic mtDNA mutations. The authors are nicely able to deomnstrate that expression of mtZFNs specific to the 4977 common single deletion of mtDNA and the m.8993T>G MTATP6 gene mutation associated with the NARP phenotype can modulate levels of mtDNA heteroplasmy, leading to a restoration of mitochondrial physiological function as determined by microscale oxygraphy.

The paper is clear, succint and very-well written. This is a logical and meaningful extension to previous proof-of principle experiments published by this group (Minczuk et al PNAS 2006) and I have only a few comments to make:

1. Abstract - minor point but the majority of patients with single, large-scale mtDNA deletions and in particular the common deletion have neither KSS or Pearson's syndrome but adult-onset chronic progressive external ophthalmoplegia (CPEO) associated with ptosis and restriction of eye movements (see recent seried reviewed by JP Grady and colleagues Brain Nov 25 2013).

2. Results - could the authors comment on the growth characteristics of cells grown in which mutation-specific mtZFN monomers were expressed as compared to controls. Any differences in doubling time, growth, viability?

3. Results - quantification of mtDNA heteroplasmy level changes.
This is a fundamental part of the data presented in the manuscript and as such the methodology used to determine mtDNA heteroplasmy levels in cells expressing mutation-specific mtZFNs needs to be robust. The assay for the m.8993T>G NARP point mutation utilises the inclusion of a 32P-

radiolabeled dNTP in a final cycle of PCR to negate effects of heteroduplex formation, with mutant and wild type genotypes discriminated on the basis of a single restriction site which is recognised in the mutant sequence. For the data shown in Figure 3C therefore, the uncut PCR product appears to be the same size as the wild type band - ie no positive control for digestion. Given the very subtle differences in mtDNA heteroplasmy being assessed, the authors should look to confirm this by other methodologies, for example pyrosequencing which is routinely used in diagnostic laboratories investigating patients with suspected mitochondrial diseae and which demonstrates a sensitivity down to 1% of mutated mtDNA.

4. Quantification of the 4977bp common deletion - as above, there are numerous qPCR assays available to accurately quantitate the level of mtDNA deletions, even down to the level of a single cell, and it would be more impressive if the authors were able to supplement the Southern blotting (with a mtDNA and nuclear probe) data with quantitative real-time PCR data to accurately determine the level of mtDNA deletion within the various treated cells. The level of sensitivity for a Southern blot can certainly be improved upon (see also data shown in Fig 4b as well as Fig 3d)

5. The data shown in Figure 5 demonstrating a recovery of mitochondrial respiratory function and capacity on shifting mtDNA heteroplasmy levels to increasing wild type levels are impressive and appear robust. The western blots however in Fig 4C, particularly for component proteins encoded by mtDNA (MTCO2) or the NDUFB8 of complex I which is a useful surrogate for the status of mtDNA subunits could be improved. Why are there 3 bands in this panel? The signal for NDUFB8 is unusually weak and it may have been helpful to investigate other marker proteins in particular additional components of cytochrome oxidase and complex I and in particular complex III (eg core 2 protein)

Referee #3 (Comments on Novelty/Model System):

This manuscript represents an important contribution as it uses mitochondrially targeted Zn-finger protein to manipulate mtDNA heteroplasmy.

Referee #3 (Remarks):

I feel this paper is at a technical high level and represents some innovative work to design mitochondrial Zn-finger nucleases so that they can be used to destroy mutant mtDNA. The strategy to use FokI variants that are only active as heterodimers is clever. The limitation of this work is of course that it is all in in vitro and that it is unclear if a similar strategy is feasible in vivo. Nevertheless, I feel paper represents a substantial step forward and provides proof of concept that Zn-fingers indeed have the potential to treat mitochondrial disease caused by specific point mutations.

I only have a few minor comments as I feel the paper is generally of high quality with solid experiments:

1. The authors add a tag (HA or Flag) immediately after the MTS. How exactly was this done to avoid that the tag interferes with the cleavage of the MTS. Did the authors use some defined spacer sequence.

2. The immunofluorescence of Fig 1C is of insuffficent quality. Individual mitochondria cannot be seen and the cytoplasm looks like a green or red blob. I would like to see confocal microscopy images of much better quality.

3. Fig. 1D. The preforms of the transfected Zn-finger nucleases are visible. This seems a bit awkward to me and indicates a very strong forced expression that may saturate the import machinery. Some comment about this would be warranted.

© EMBO

1st Revision - authors' response 06 January 2014

Referee #1 (Remarks): 'This paper describes a significant improvement in the ZFN approach to selectively degrading mutated mtDNA.
I have relatively few comments:

1. One of my main concerns is the relative inefficiency of the ZFN approach to change the level of heteroplasmy in the m.8993T>C cells - there is a relatively small shift in heteroplasmy and much less than in the single deletion cell lines. Is there an explanation for this and do the authors feel this will reduce the utility of this approach. '

This is an interesting and valid point. It is our belief that a smaller shift in heteroplasmy is observed in the m.8993T>G cell line because there is a far lower starting quantity of wild-type mtDNA in the m.8993T>G cell line (<7%), especially when compared with the 'common deletion' cell line (16%). Repopulation of wild-type mtDNA might, therefore, be diminished after an initial, rapid reduction of mtDNA copy-number. For example, extensive cell death could occur upon drastically reduced mtDNA content, or temporal insufficiency of TFAM, a factor necessary for mtDNA replication, might occur (it is known that cells depleted of mtDNA contain a much reduced steady-state level of TFAM - Seidel-Rogol and Shadel 2002, Nucleic Acids Res. 30:1929-34). Currently we are testing the hypothesis of whether higher initial loads of wild-type mtDNA would result in more efficient heteroplasmy shifts, however we feel that these experiments are beyond the scope of this manuscript. If this hypothesis is true, several sequential rounds of transfection with mtZFNs could totally eliminate the mutant mtDNA. In addition to this, and as noted in the main text (page 15) of the manuscript, the m.8993T>G cell line has a tendency to shift heteroplasmy towards the mutant haplotype (to ~3%) while in culture (Fig. 2D), exacerbating the effect of copy number depletion as mentioned previously.

We have added the following paragraph to the Discussion:
"The highly efficient shift in heteroplasmy from mutant to wild-type for the CD cell line contrasted with the shift observed in the m.8993T>G cells. There are two plausible reasons for this: (i) there is a far lower starting quantity of wild-type mtDNA in the m.8993T>G cell line, therefore the rapid initial elimination of mutant mtDNA leaves very little capacity for mtDNA repopulation. (ii) The m.8993T>G cell line has a tendency to shift heteroplasmy towards the mutant haplotype (Fig. 2D), both masking the efficacy of mtZFNs and exacerbating the previously stated effect. Further research is in progress in our laboratory to verify the hypothesis that a higher initial load of wild-type mtDNA would result in more efficient heteroplasmy shifts of m.8993T>G by mtZFNs. If this assumption is correct, several sequential transfections could produce complete (or near complete) elimination of mutant mtDNA."

2. 'As far as I can tell the authors do not specifically say how long they left the cells after transfection/selection - just wondered if they had varied this to see if they get better loss of mutated mtDNA. Is it possible to completely remove the mutated form?'

It has been noted in the Materials & Methods section of the manuscript that cells were sequentially transfected and selected with plasmids encoding one mtZFN construct, and one of two antibiotic resistance cassettes, for 18 days each, after which they were harvested and analysed. Therefore, the cells were exposed to both mtZFN monomers, simultaneously, for ~18 days. We allowed the cells to grow in culture with both mtZFN monomers present for up to 34 days , but saw no significant further changes in heteroplasmy beyond the initial measurements. As already mentioned in point 1, experiments are in progress to assess whether several sequential transfections, where cells are exposed to mtZFNs for a brief period, will impact on this. However, we feel that these experiments are beyond the scope of this manuscript, presenting the proof-of-principle for using our improved obligatory heterodimeric mtZFNs.

We have changed the text as follows:
"Simultaneous expression of NARPd(+) and COMPa(-) for 18 days, as assessed by western blotting, resulted in selective degradation of the m.8993T>G mtDNA molecules, which was accompanied by a shift in heteroplasmy of the cybrids from ~7% wild-type to ~17% wild-type, as

assessed by RFLP (Fig. 2c and 2d)."
and
"We analysed mtDNA heteroplasmy in transfected H39 cells using Southern blotting (Fig. 3d). This analysis showed that simultaneous expression of both the R8-4(+) and R13-1(-) mtZFNs for 18 days led to efficient degradation of CD mtDNA, shifting the proportion of wild-type mtDNA from ~15% to ~76% (Fig. 3d)."

3. 'Figure 4c is somewhat confusing - the WT there are three bands but only two proteins. Also the ratio of CO2 to NDUFB8 is very different from the cells treated with the ZFNs'

The referee raised some concerns regarding the identity of bands observed in western blot of OXPHOS subunits. The band seen below NDUFB8 is presumed to be non-specific or a product of partial proteolysis of the sample. In response to the reviewers comment, we have produced a new and improved set of blots from these samples (revised Fig. 4c). We, additionally, included lysates of isolated mitochondria from HOS 143B and CD cells to help indicate the bands of interest.

The Reviewer has also pointed out that the ratio of CO2 to NDUFB8 is altered in mtZFN treated cells. We agree with the Reviewer. We would tentatively suggest that this is due to cells, which have been cultured for a significant length of time with selective drugs, adapting to their environment. Alternately, this could be an artefact of the cybrid model itself. Nonetheless, mtZFN treated cells show much higher levels of CO2 and NDUFB8 ,which is the key conclusion from this experiment.

Referee #2 (Comments on Novelty/Model System): Appropriate cellular models used in proof of principle experiments

Referee #2 (Remarks): The paper by Gammage et al investigates the use of mitochondrially-targeted zinc-finger nucleases (mtZFNs) to human cells harbouring pathogenic mtDNA mutations. The authors are nicely able to demonstrate that expression of mtZFNs specific to the 4977 common single deletion of mtDNA and the m.8993T>G MTATP6 gene mutation associated with the NARP phenotype can modulate levels of mtDNA heteroplasmy, leading to a restoration of mitochondrial physiological function as determined by microscale oxygraphy. The paper is clear, succinct and very-well written. This is a logical and meaningful extension to previous proof-of principle experiments published by this group (Minczuk et al PNAS 2006) and I have only a few comments to make:

1. Abstract - minor point but the majority of patients with single, large-scale mtDNA deletions and in particular the common deletion have neither KSS or Pearson's syndrome but adult-onset chronic progressive external ophthalmoplegia (CPEO) associated with ptosis and restriction of eye movements (see recent seried reviewed by JP Grady and colleagues Brain Nov 25 2013).

We would like to thank the reviewer for bringing this to our attention. An amendment has been made to the abstract and the main text, with the citation to the paper mentioned by the Reviewer. The text changes as follows:

Abstract: "the 'common deletion' (CD), a 4977bp repeat-flanked deletion associated with adult-onset chronic progressive external ophthalmoplegia and, less frequently, Kearns-Sayre and Pearson's marrow pancreas syndromes."

Result section: "CD often manifests in adult-onset chronic progressive external ophthalmoplegia (CPEO) associated with ptosis and restriction of eye movements, and, less frequently, with Kearns-Sayre and Pearson's marrow pancreas syndromes (Grady, et al 2013)."

2. Results - could the authors comment on the growth characteristics of cells grown in which mutation-specific mtZFN monomers were expressed as compared to controls. Any differences in doubling time, growth, viability?

In contrast to our previous designs of mtZFNs containing unmodified *Fok*I domain (Minczuk et al. Nucleic Acids Res. 2008 36:3926-38), the current design encompassing the inactive obligatory heterodimeric nuclease did not show any appreciable cell toxicity. This was measured by cotransfecting an equal number of cells with plasmids containing either fluorescent markers only, or fluorescent markers and mtZFN in different combinations, followed by analysing the percentage of marker positive cells by flow cytometry (approach used previously in Minczuk et al. 2008)

This is also supported by the experiment presented in Suppl. Fig S3, where we show that the presence of a mtZFN monomer in the mitochondrial matrix did not produce any appreciable effect on mtDNA replication.

Also, as mentioned in the manuscript, we did observe an increase in growth rate in clonal CD cell lines where heteroplasmy had been shifted towards wild-type mtDNA by mtZFN (Suppl. Figure S6).

We have added an appropriate comment to the text:

"Expression of NARPd(+) and/or COMPa(-) did not have any significant effect on mtDNA copy number (Supporting Information Fig. S2) or cell viability, as informed by cotransfecting equal numbers of cells with mtZFN monomers and fluorescent markers, followed by determining the percentage of marker positive cells with a flow cytometer (Supporting Information Table S1)"

We also present the flow cytometry data in Supporting. Table S1.

3. Results - quantification of mtDNA heteroplasmy level changes. This is a fundamental part of the data presented in the manuscript and as such the methodology used to determine mtDNA heteroplasmy levels in cells expressing mutation-specific mtZFNs needs to be robust. The assay for the m.8993T>G NARP point mutation utilises the inclusion of a 32P-radiolabeled dNTP in a final cycle of PCR to negate effects of heteroduplex formation, with mutant and wild type genotypes discriminated on the basis of a single restriction site which is recognised in the mutant sequence. For the data shown in Figure 3C therefore, the uncut PCR product appears to be the same size as the wild type band - i.e. no positive control for digestion. Given the very subtle differences in mtDNA heteroplasmy being assessed, the authors should look to confirm this by other methodologies, for example pyrosequencing which is routinely used in diagnostic laboratories investigating patients with suspected mitochondrial disease and which demonstrates a sensitivity down to 1% of mutated mtDNA.

The last-cycle-hot PCR assay used to detect m.8993T>G mtDNA in these experiments uses the '99% m.8993T>G Control DNA' as a positive control for digestion (Fig. 2c) and is able to detect as little as 1% wild-type mtDNA. Such sensitivity is similar to the diagnostic method referenced by the Reviewer. Furthermore, the experiments in question have been performed three times, the difference between the controls and experimental samples was statistically significant and the level of heteroplasmy over 5 times higher between the experiment and control.

In response to the Referee's concerns we have included Supporting. Fig. S1 in the revised version of the manuscript. This figure presents a careful validation of our method using cell lines bearing different m.8993T>G mutant loads. Importantly, during validation of the assay we have used a sample with 100% mutant mtDNA, showing full digestion of the PCR product derived from this cell line, and providing the requested positive control for the assay. We hope that the new data provided will improve the reviewer's confidence in the robustness of our assay.

4. Quantification of the 4977bp common deletion - as above, there are numerous qPCR assays available to accurately quantitate the level of mtDNA deletions, even down to the level of a single cell, and it would be more impressive if the authors were able to supplement the Southern blotting (with a mtDNA and nuclear probe) data with quantitative real-time PCR data to accurately

determine the level of mtDNA deletion within the various treated cells. The level of sensitivity for a Southern blot can certainly be improved upon (see also data shown in Fig 4b as well as Fig 3d)

The primary reason for using Southern blotting, rather than qPCR, to measure mtDNA heteroplasmy in CD cell lines was to verify whether any deleted or recombined molecules are generated in our experiments as a result of nucleolytic cleavage of mtDNA. It has been shown previously that recombined mtDNA species can occur upon nucleolytically-induced DNA double-strand breaks in the mitochondrial genome (Bacman et al. 2009 Nucleic Acids Res. 37:4218-26). This Southern blotting approach has been established in our lab, is used routinely and allowed detecting rearranged mtDNA in other studies. However, we have not detected any recombined genomes in our mtZFN experiments.

Southern blotting measurement of mtDNA is considered as very accurate, however it requires a relatively large amount of DNA, involves several time consuming steps and usually requires use of radioactive material. These factors, in particular the availability of DNA e.g. from muscle biopsies, are likely reasons why real-time quantitative PCR (qPCR) is routinely used in clinical diagnostics. However, some features of Southern blotting can be considered superior compared to qPCR. For example, simultaneous quantification of full-length and deleted mtDNA molecules and the lack of amplification steps.

We feel that the clear, large shifts in heteroplasmy that are visible in our autoradiograms, for over 40 samples tested (Supporting Fig. S5), serve as sufficient analysis for a technology at this proof-of-principle stage.

We have amended the text to read as follows:

"We analysed mtDNA heteroplasmy in transfected H39 cells using Southern blotting, allowing detection of expected mtDNA haplotypes, as well as potential, undesired recombined mtDNA species (Bacman et al., 2009)"

and

"Mock-transfected cells or cells expressing high levels of either R8-4(+) or R13-1(-) individually did not show any appreciable changes in heteroplasmy levels and no recombined mtDNA species were detected in any conditions (Fig. 3d)."

5. The data shown in Figure 5 demonstrating a recovery of mitochondrial respiratory function and capacity on shifting mtDNA heteroplasmy levels to increasing wild type levels are impressive and appear robust. The western blots however in Fig 4C, particularly for component proteins encoded by mtDNA (MTCO2) or the NDUFB8 of complex I which is a useful surrogate for the status of mtDNA subunits could be improved. Why are there 3 bands in this panel? The signal for NDUFB8 is unusually weak and it may have been helpful to investigate other marker proteins in particular additional components of cytochrome oxidase and complex I and in particular complex III (e.g. core 2 protein)

In response to this comment we would like to refer back to our detailed response to point 3 from Reviewer 1. We have produced a new set of western blots (revised Fig. 4c) from these samples and included the antibodies to core 2 protein of complex III (UQCRC2), as well as further blotting against NDUFB8 and CO2, with isolated mitochondrial lysates to help indicate the bands of interest. We consider the band below NDUFB8 seen previously as non-specific or a product of partial proteolysis.

Referee #3 (Comments on Novelty/Model System): This manuscript represents an important contribution as it uses mitochondrially targeted Zn-finger protein to manipulate mtDNA heteroplasmy.

Referee #3 (Remarks): I feel this paper is at a technical high level and represents some innovative work to design mitochondrial Zn-finger nucleases so that they can be used to destroy mutant mtDNA. The strategy to use FokI variants that are only active as heterodimers is clever. The limitation of this work is of course that it is all in in vitro and that it is unclear if a similar strategy is feasible in vivo. Nevertheless, I feel paper represents a substantial step forward and provides proof of concept that Zn-fingers indeed have the potential to treat mitochondrial disease caused by specific point mutations. I only have a few minor comments as I feel the paper is generally of high quality with solid experiments:

1. The authors add a tag (HA or Flag) immediately after the MTS. How exactly was this done to avoid that the tag interferes with the cleavage of the MTS. Did the authors use some defined spacer sequence.

This MTS sequence was designed using data taken from Carroll et al (2009, Anal. Biochem.), who used mass spectrometric methods to analyse purified subunits of ATP synthase. Their analysis suggested that the latest start site for the mature, processed F1b subunit of ATP synthase is Ala48. The F1b MTS used in the mtZFN construct includes sequence from Met1 up to Gln49 of F1b, ensuring that cleavage of the MTS occurs downstream of the epitope tag. We have added an appropriate comment to the text:

Mitochondrial targeting is facilitated by 49 amino acid-long MTS from subunit F1β of human mitochondrial ATP synthase. To ensure that the MTS cleavage site is downstream of the epitope tag, its length was adjusted using proteomic data (Carroll et al., 2009) that identified the N-terminus of the mature subunit at Ala48.

2. The immunofluorescence of Fig 1C is of insufficient quality. Individual mitochondria cannot be seen and the cytoplasm looks like a green or red blob. I would like to see confocal microscopy images of much better quality.

Following the Reviewer's recommendation, we have improved the microscopy images that are presented in revised Fig. 1C.

3. Fig. 1D. The preforms of the transfected Zn-finger nucleases are visible. This seems a bit awkward to me and indicates a very strong forced expression that may saturate the import machinery. Some comment about this would be warranted.

We agree with the reviewer on this point. It has been observed previously that high expression of proteins (such as that driven by a high expression promoter, such as CMV, used in this study) can lead to saturation of the mitochondrial import machinery. We have included a comment to this effect:

" This experiment also showed that the MTS is cleaved-off from precursor mtZFN proteins, although incompletely; this phenomenon is often observed, even for endogenous mitochondrial proteins expressed at a high level (Maniura-Weber et al.2004)."

2nd Editorial Decision 08 January 2014

Thank you for the submission of your revised manuscript to EMBO Molecular Medicine. I am pleased to inform you that we will be able to accept your manuscript in principle, pending final editorial amendments.
