# Supplementary figures and images for: Mitochondrially targeted ZFNs for selective degradation of pathogenic mitochondrial genomes bearing large-scale deletions or point mutations
Source: EMBO Mol Med. 2014 Feb 24;6(4):458–66. doi: 10.1002/emmm.201303672 (PMC3992073; doi:10.1002/emmm.201303672)

Figure 1 - Panel D

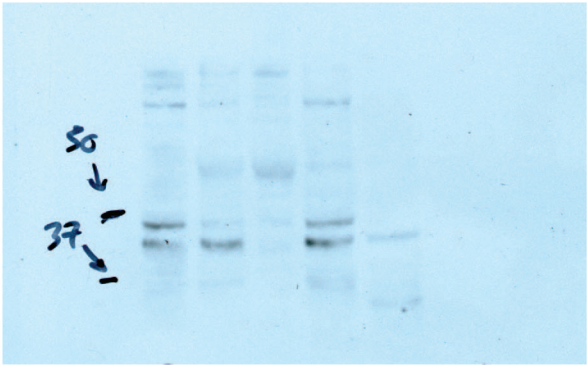

anti-HA

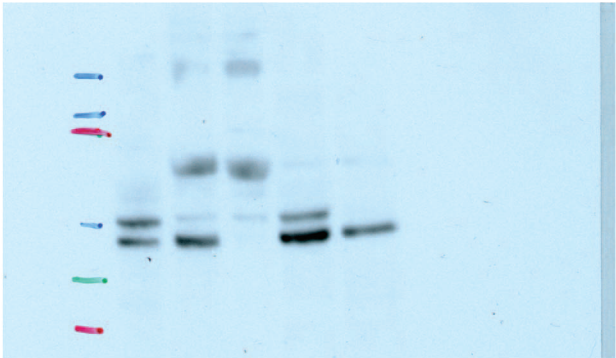

anti-FLAG

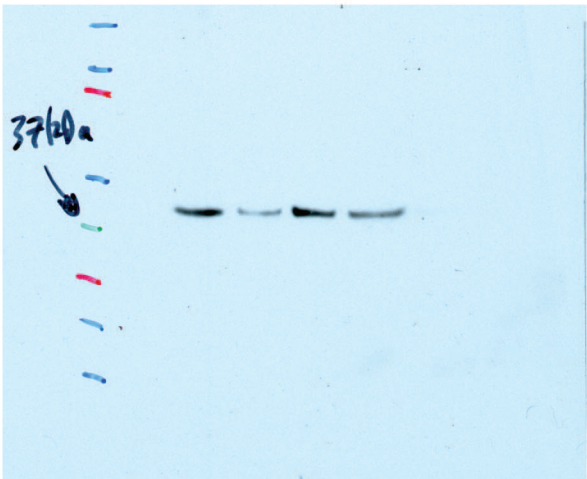

anti-B-actin

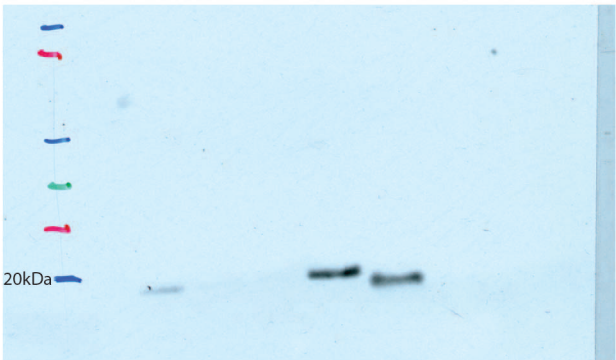

anti-TOM22

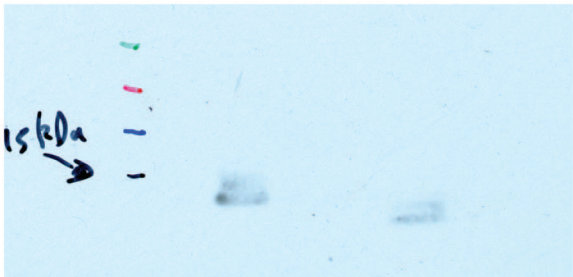

anti-H4

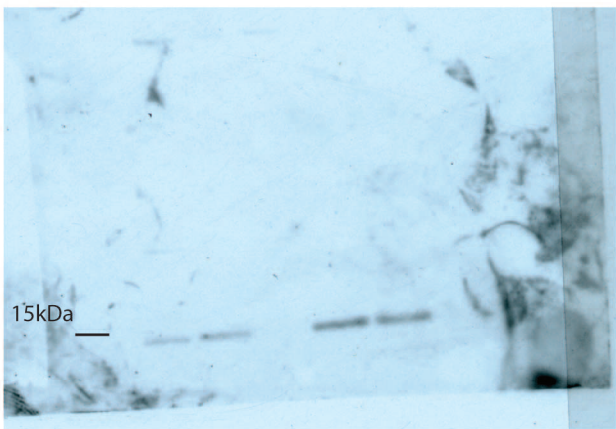

anti-SSB1

Supplement: Supplementary file 2 [file emmm0006-0458-sd2.pdf]

Figure 3 - Panel C

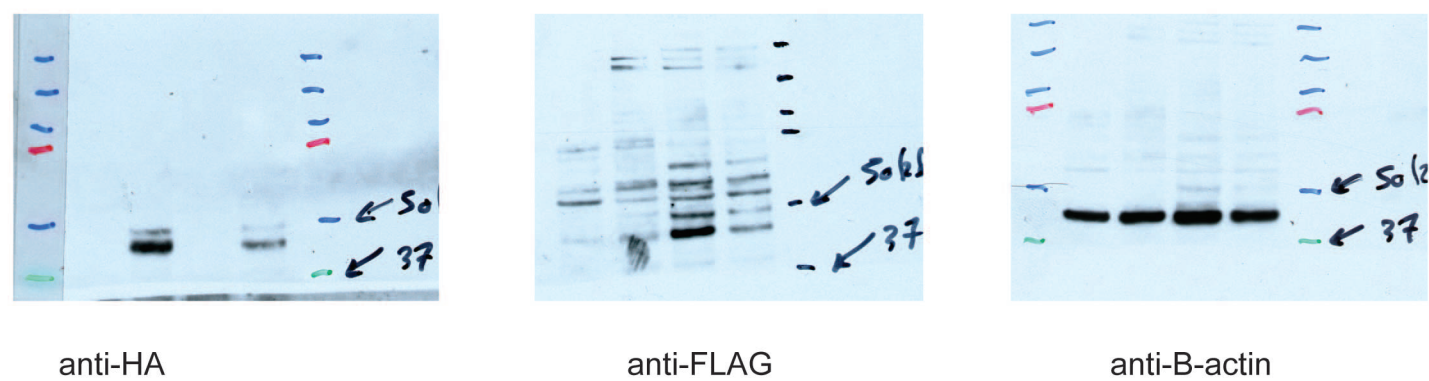

Figure 3 - Panel D

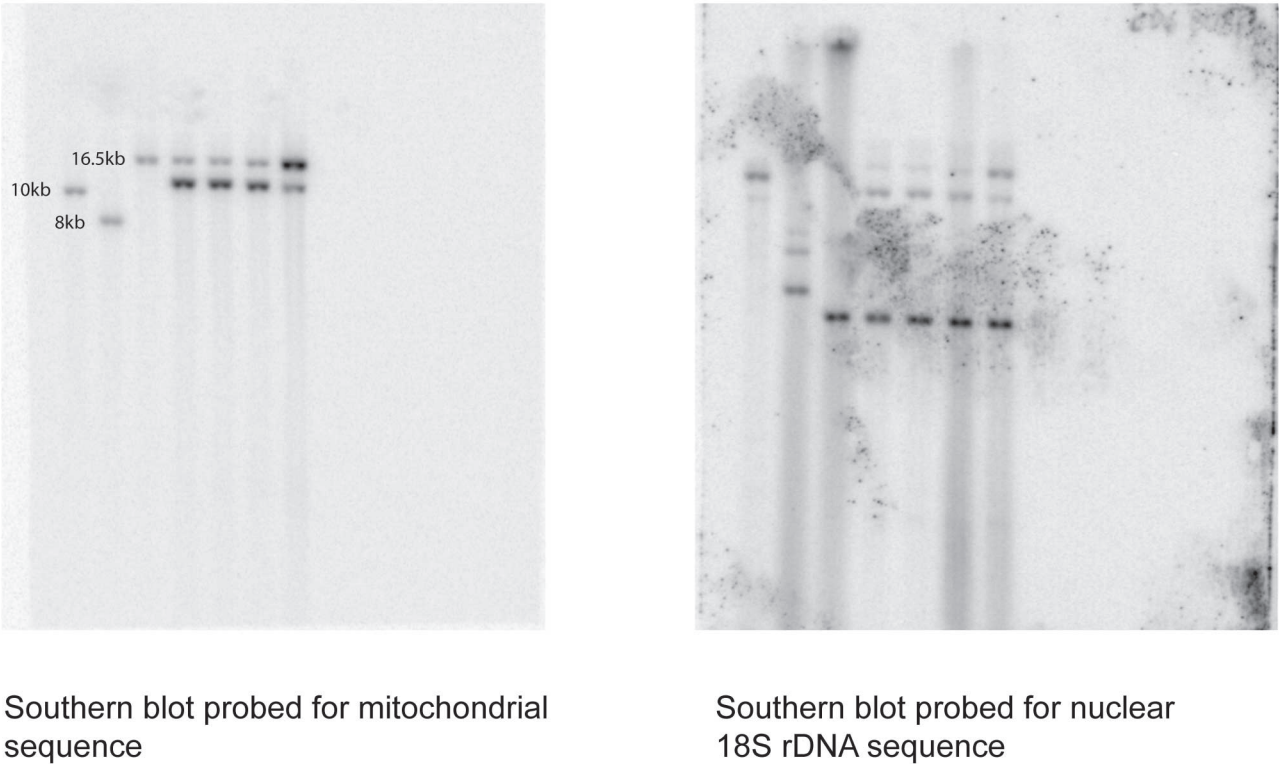

Supplement: Supplementary file 4 [file emmm0006-0458-sd4.pdf]
