# Supplementary material for: Mitochondrially targeted ZFNs for selective degradation of pathogenic mitochondrial genomes bearing large-scale deletions or point mutations
Source: EMBO Mol Med. 2014 Feb 24;6(4):458–66. doi: 10.1002/emmm.201303672 (PMC3992073; doi:10.1002/emmm.201303672)
Supplement: Supplementary file 3 [file emmm0006-0458-sd3.pdf]

Figure 2 - Source data

Source data - Gammage *et al.*

## Figure 2 - Panel B

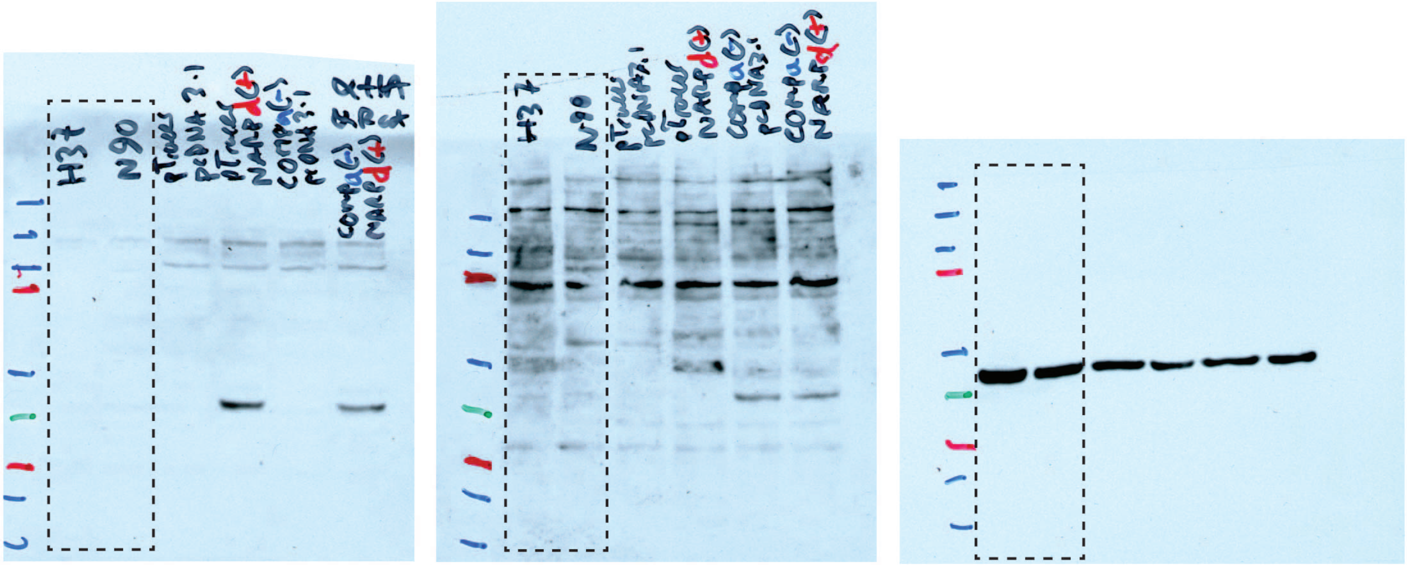

anti-HA anti-FLAG anti-B-actin

Dashed boxes contain untransfected controls that aren’t included in Figure 2

## Figure 2 - Panel C

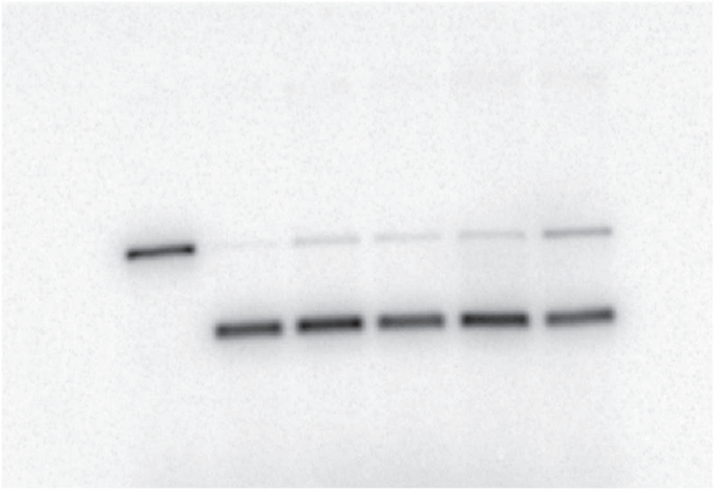

Image from storage phosphor screen

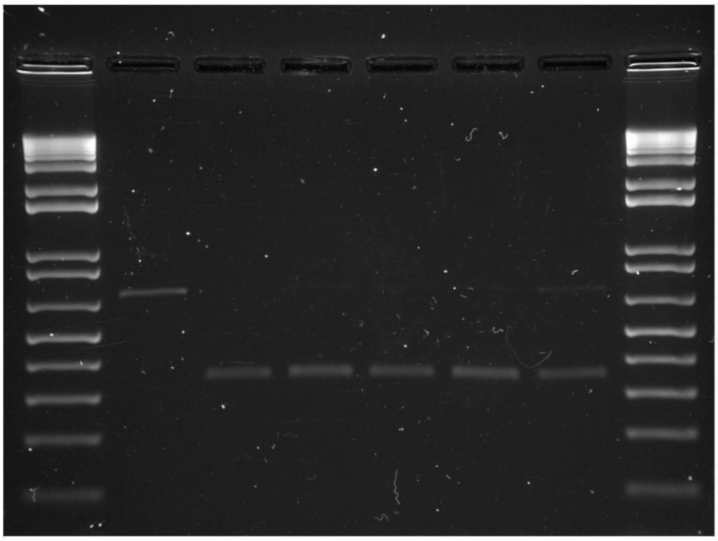

UV image prior to gel drying

Invitrogen 1kb plus ladder
