# Supplementary material for: Mitochondrially targeted ZFNs for selective degradation of pathogenic mitochondrial genomes bearing large-scale deletions or point mutations
Source: EMBO Mol Med. 2014 Feb 24;6(4):458–66. doi: 10.1002/emmm.201303672 (PMC3992073; doi:10.1002/emmm.201303672)
Supplement: Supplementary file 5 [file emmm0006-0458-sd5.pdf]

Figure 4 - Panel B

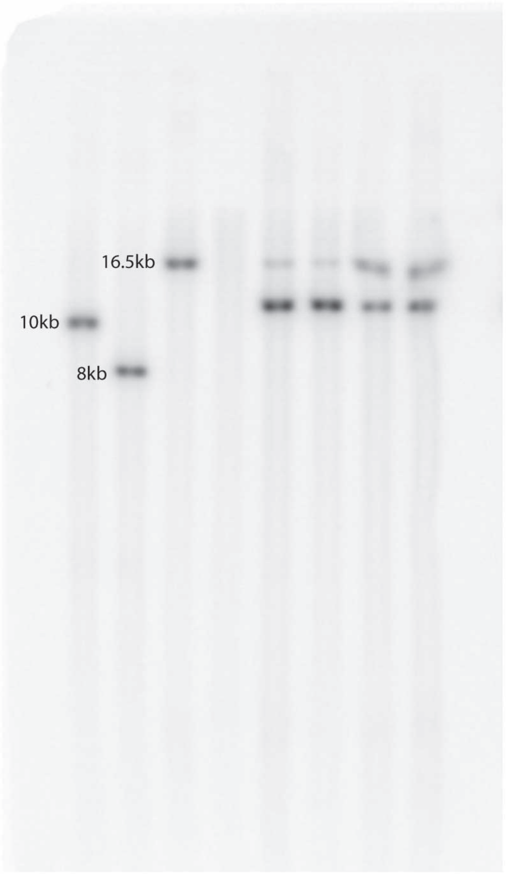

Southern blot probed for mitochondrial sequence

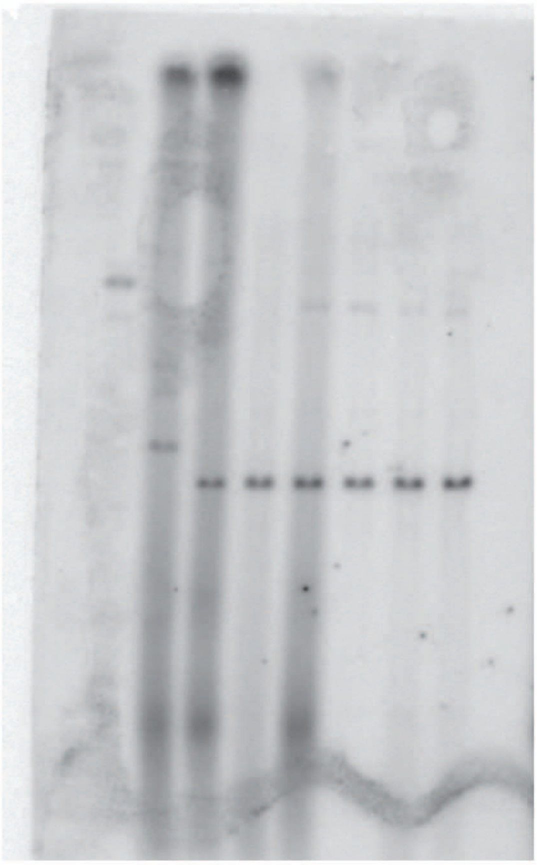

Southern blot probed for nuclear 18S rDNA sequence

Figure 4 - Panel C

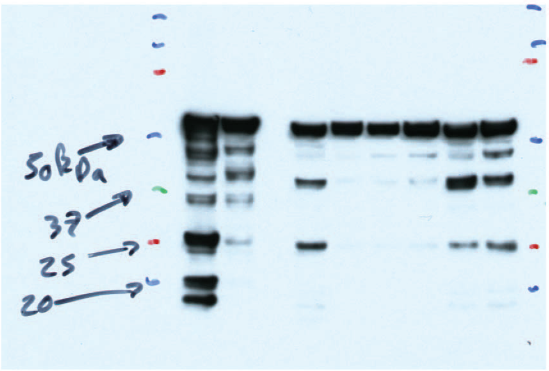

anti-OXPHOS cocktail

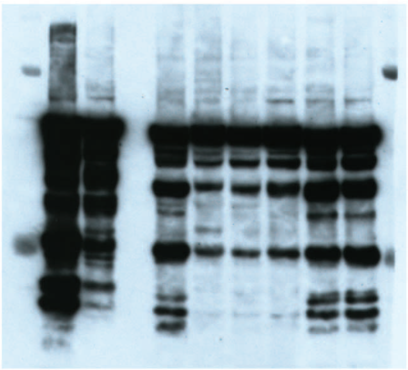

anti-OXPHOS cocktail
Longer exposure

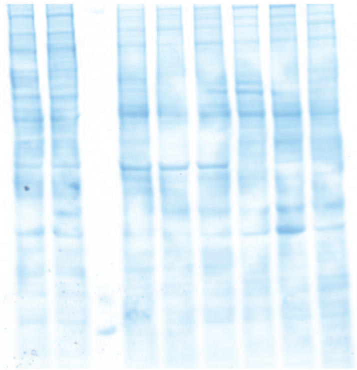

Coomassie staining
