# Supplementary material for: Mitochondrially targeted ZFNs for selective degradation of pathogenic mitochondrial genomes bearing large-scale deletions or point mutations
Source: EMBO Mol Med. 2014 Feb 24;6(4):458–66. doi: 10.1002/emmm.201303672 (PMC3992073; doi:10.1002/emmm.201303672)
Supplement: Supplementary file 6 [file emmm0006-0458-sd6.pdf]

Supporting Figure S1: Validation of RFLP last cycle hot (LCH) PCR assay

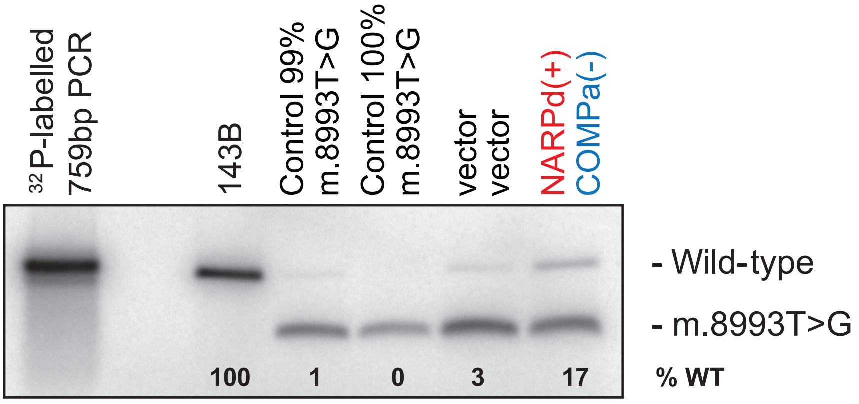

To ensure the accuracy of our RFLP LCH PCR assay, we amplified and digested mtDNA from samples of previously measured mtDNA heteroplasmy (lanes 6,7 included in Fig. 2D of the main text) and controls. Complete preservation of 143B DNA and complete digestion of 100% m.8993T>G DNA demonstrate the efficiency of *Sma*I digestion. Preservation of wild-type mtDNA in 99% m.8993T>G DNA confirms the accuracy of this method for determining mtDNA heteroplasmy to ~1%.
