# Supplementary material for: Mitochondrially targeted ZFNs for selective degradation of pathogenic mitochondrial genomes bearing large-scale deletions or point mutations
Source: EMBO Mol Med. 2014 Feb 24;6(4):458–66. doi: 10.1002/emmm.201303672 (PMC3992073; doi:10.1002/emmm.201303672)
Supplement: Supplementary file 7 [file emmm0006-0458-sd7.pdf]

Supporting Figure S2: Mitochondrial DNA copy number in cybrid cells transfected with m.8993T>G-specific mtZFNs

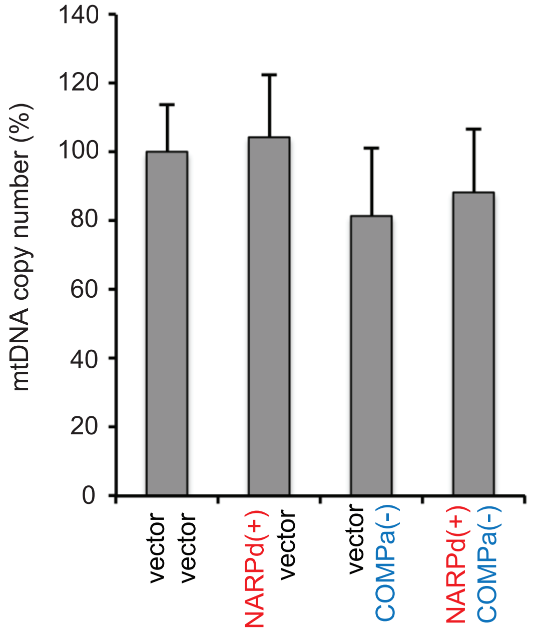

Comparative Southern blot analysis of the mitochondrial genome (probe position mt. 14986-15607) and 18S rDNA with ImageQuant software (GE Healthcare) was performed using total DNA isolated from bulk populations of cells expressing m.8993T>G-specific mtZFNs NARPd and COMPa, and appropriate control vectors. n = 3, Error bars = 1 SD.
