# Supplementary material for: Mitochondrially targeted ZFNs for selective degradation of pathogenic mitochondrial genomes bearing large-scale deletions or point mutations
Source: EMBO Mol Med. 2014 Feb 24;6(4):458–66. doi: 10.1002/emmm.201303672 (PMC3992073; doi:10.1002/emmm.201303672)
Supplement: Supplementary file 8 [file emmm0006-0458-sd8.pdf]

Supporting Figure S3: Two-dimensional agarose gel electrophoresis (2D-AGE) of mtDNA from HOS 143B cells expressing mtZFN binding to wild-type sequence

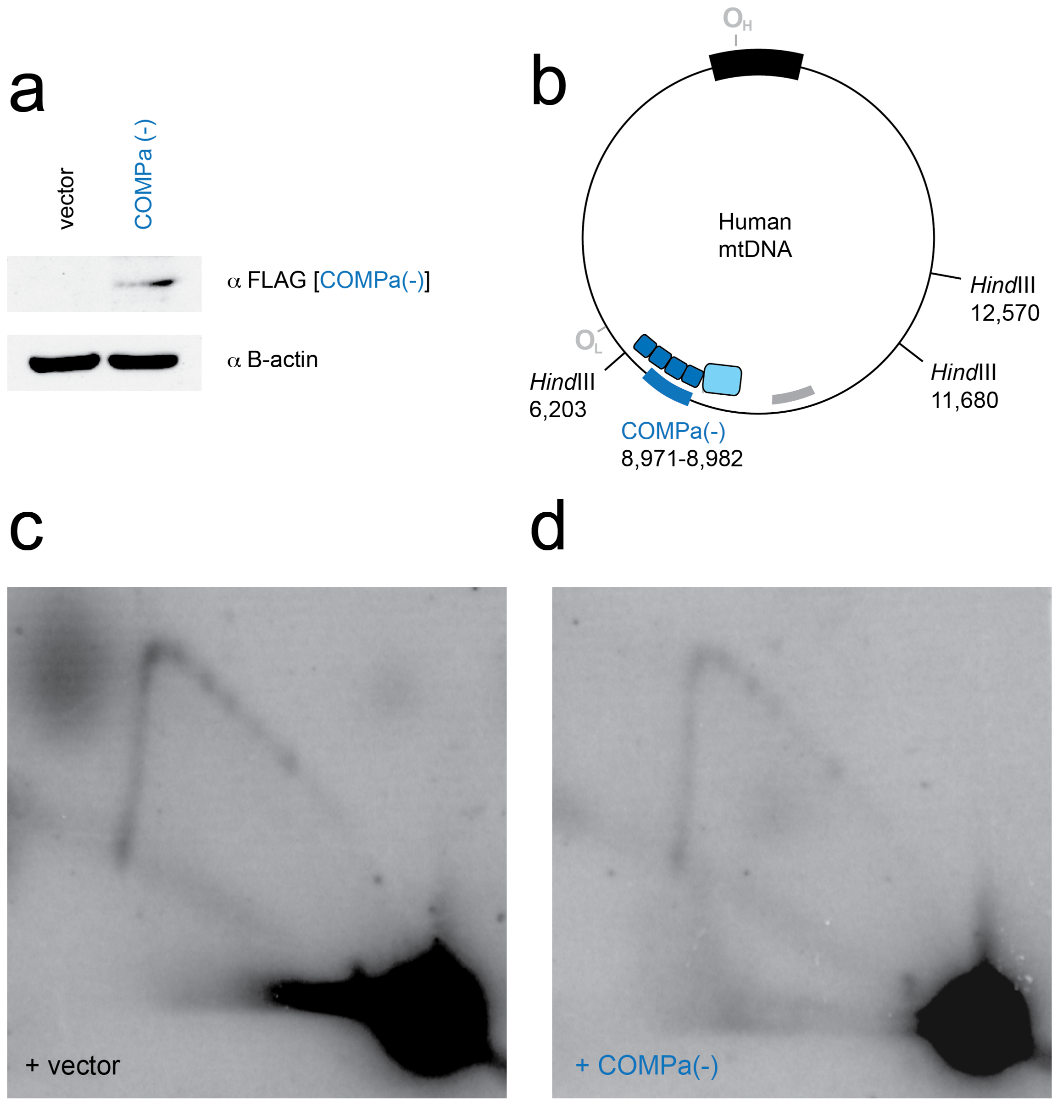

(A) Western blot analysis of HOS 143B cells stably expressing either the COMPa(-) mtZFN targeted to wild-type mtDNA sequence or a control vector. (**B**) Schematic representation of restriction digest used in the 2D-AGE analysis. The COMPa(-) mtZFN binding site is indicated by a blue block. The probe used for Sothern blotting is indicated by a grey block. (**C**) and (**D**) 2D-AGE followed by Southern blot analysis of replication intermediates in mock transfected HOS 143B cells (C) or cell stably expressing COMPa(-) mtZFN (D).
