# Supplementary material for: Mitochondrially targeted ZFNs for selective degradation of pathogenic mitochondrial genomes bearing large-scale deletions or point mutations
Source: EMBO Mol Med. 2014 Feb 24;6(4):458–66. doi: 10.1002/emmm.201303672 (PMC3992073; doi:10.1002/emmm.201303672)
Supplement: Supplementary file 9 [file emmm0006-0458-sd9.pdf]

Supporting Figure S4: *In vitro* testing of ZFPs specific to CD

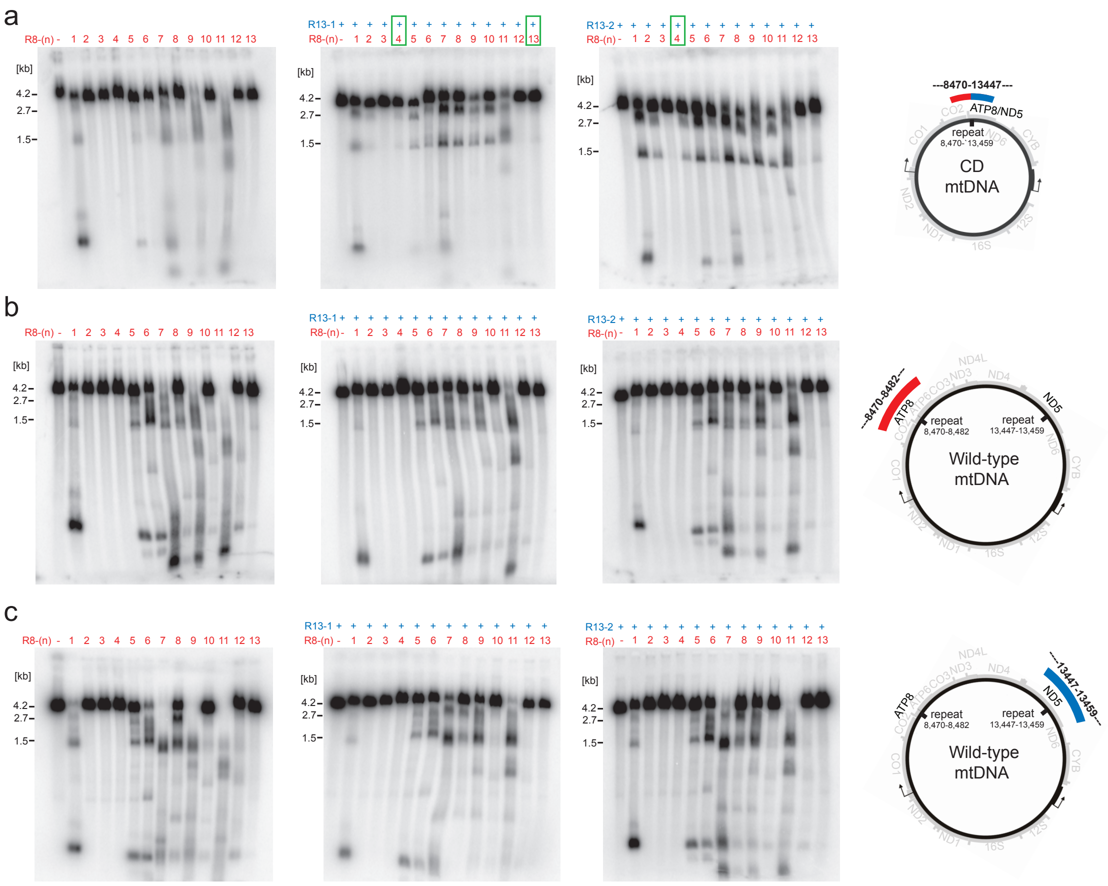

In vitro assay testing the specificity of the R8-*n* and R13-*n* constructs for the CD site. The assay was performed with *in vitro* translated ZFNs as described in detailed protocols published previously (Minczuk *et al.*, 2010). Uncleaved DNA substrate: 4.2 kb, specific cleavage products: 2.7 + 1.5 kb. The left hand column shows the results with the R8-*n* constructs alone. The middle and the right hand side columns show the activity of different combinations of the R8-*n* and the R13-*n* constructs. Wild-type, rather than obligatory heterodimer, *Fok*I was used in this experiment in order to reveal any DNA cleavage events due to non specific binding and/ or homodimerization of ZFN monomers.

(A) *In vitro* cleavage assay for the ---8470-13447--- probe corresponding to the joining site resulting from CD. **(B)** Results obtained for the ---8470-8482--- probe corresponding to the mtDNA wild-type sequence in the vicinity of the 8470-8482 direct repeat. **(C)** Results obtained for the ---13447-13459--- probe corresponding to the wild-type sequence surrounding the 13447-13459 repeat. The sequences of the probes and the design are given in **Supporting Text**. The regions of human mtDNA contained within the probes used for the *in vitro* assay are schematically presented on the right-hand side of the figure.

Green boxes indicate the pairs of mtZFNs tested in cybrid cells, as described in the main text.
