# Supplementary material for: Mitochondrially targeted ZFNs for selective degradation of pathogenic mitochondrial genomes bearing large-scale deletions or point mutations
Source: EMBO Mol Med. 2014 Feb 24;6(4):458–66. doi: 10.1002/emmm.201303672 (PMC3992073; doi:10.1002/emmm.201303672)
Supplement: Supplementary file 10 [file emmm0006-0458-sd10.pdf]

Supporting Figure S5: Expression of mtZFNs, mtDNA heteroplasmy and mtDNA copy number in clonal cybrid cells transfected with common deletion-specific mtZFNs

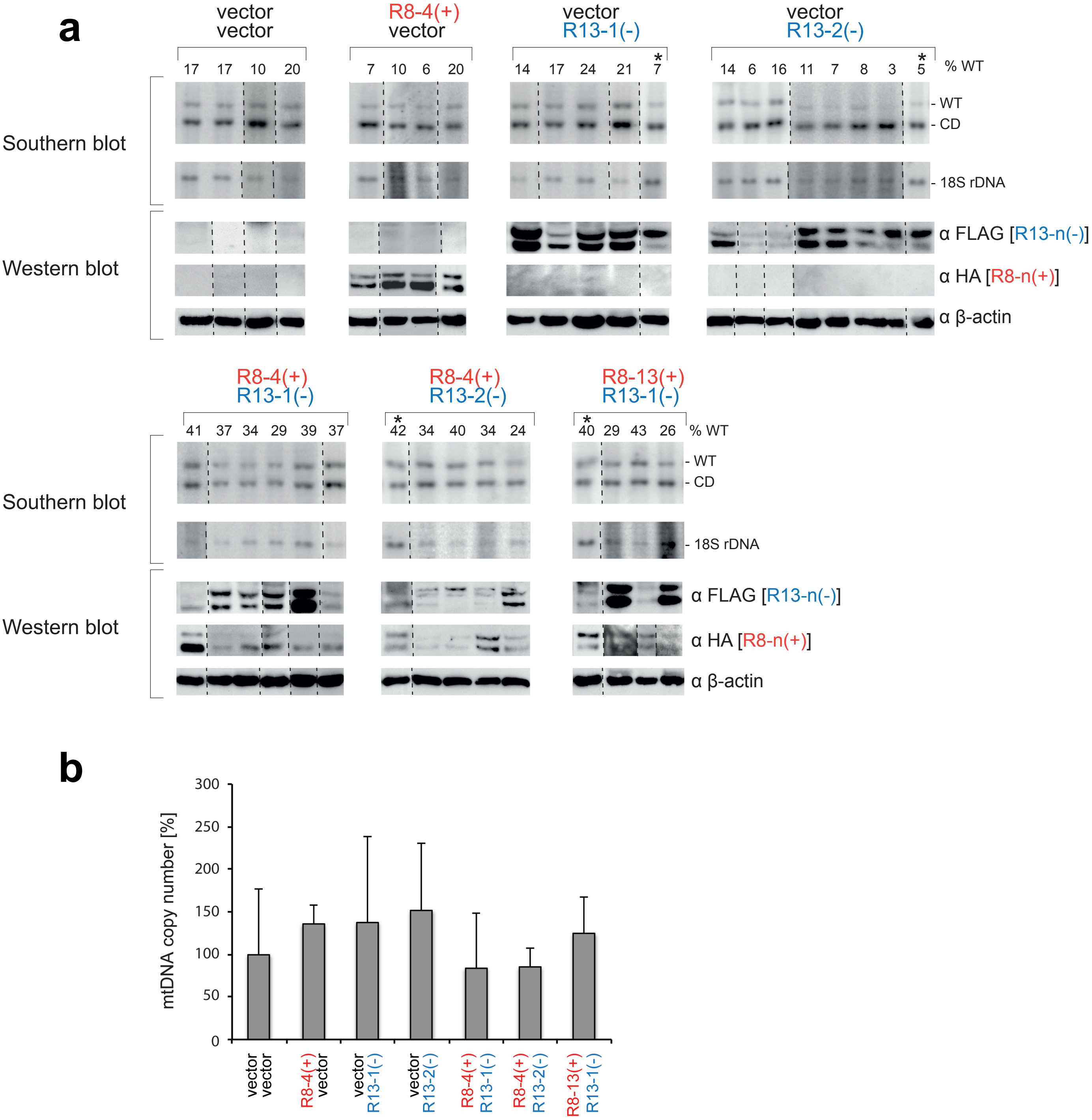

(A) Analysis of clones transfected with CD-specific mtZFN constructs, and appropriate control vectors. Presented (from top) are Southern blot (mtDNA, 18S rDNA) and western blot (HA, FLAG, β-actin) analyses from all clones presented in **Fig. 3E** of the main text. Asterisks indicate clones used for the phenotype rescue experiments in **Fig. 4** of the main text. Percentage of wild-type (WT) mtDNA is given above each lane.

Supporting Figure S5 (continued)

(B) Relative quantification of Southern blot analyses of the mitochondrial genome (probe position mt. 14986-15607) and 18S rDNA, performed using ImageQuant software (GE Healthcare). Total cellular DNA was isolated from clones expressing common deletion-specific mtZFNs and control vectors. Southern blotting and probes were as described in Material and Methods in the main text. $n \geq 4$, Error bars = 1 SD.
