# Supplementary material for: Mitochondrially targeted ZFNs for selective degradation of pathogenic mitochondrial genomes bearing large-scale deletions or point mutations
Source: EMBO Mol Med. 2014 Feb 24;6(4):458–66. doi: 10.1002/emmm.201303672 (PMC3992073; doi:10.1002/emmm.201303672)
Supplement: Supplementary file 11 [file emmm0006-0458-sd11.pdf]

Supporting Figure S6: Growth rate of mtZFN-transfected CD cybrid cells

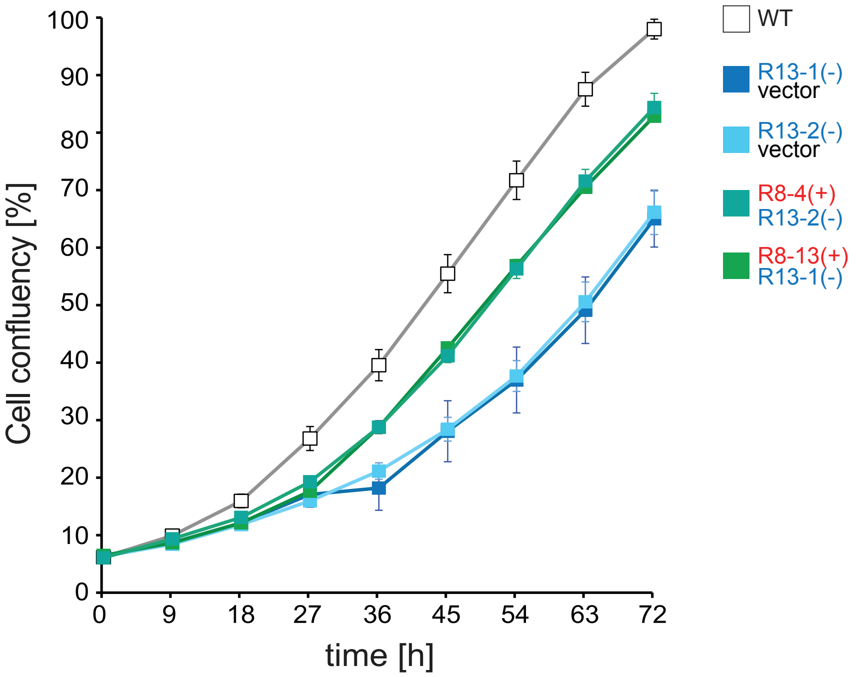

Growth rate expressed as cell confluence measured by the INCUCYTE™ Kinetic Imaging System (Essen Bioscience) in triplicate for wild-type (WT, white) and clonal cells expressing CD-specific mtZFNs and appropriate control vectors from the same transfection.
