# Supplementary material for: Mitochondrially targeted ZFNs for selective degradation of pathogenic mitochondrial genomes bearing large-scale deletions or point mutations
Source: EMBO Mol Med. 2014 Feb 24;6(4):458–66. doi: 10.1002/emmm.201303672 (PMC3992073; doi:10.1002/emmm.201303672)
Supplement: Supplementary file 12 [file emmm0006-0458-sd12.pdf]

Supporting Table S1: Flow cytometry analysis of cells transfected with mtZFNs

	% live cells	% dual transfected (of total)
**mock**<br>**mock**	65.8	27.2 (17.3)
**NARPd(+)**<br>**mock**	59.1	23.6 (13.5)
**mock**<br>**COMPa(-)**	63.2	25.3 (15.4)
**NARPd(+)**<br>**COMPa(-)**	66.9	24.6 (16)

To assess whether mtZFN constructs effect on cell viability, we co-transfected equal numbers of NARP m.8993T>G cells with plasmids encoding either NARPd (+)/mCherry or COMPa(-)/GFP, or empty vectors in all possible combinations. Cells were analysed 24 hours post-transfection with a flow cytometer, and percentages of total live cells and dual-transfected live cells were calculated. No obvious differences were observed between the conditions, suggesting that no cytotoxic effect is exerted by mtZFN expression or nucleolytic cleavage.
