# Supplementary material for: Mitochondrially targeted ZFNs for selective degradation of pathogenic mitochondrial genomes bearing large-scale deletions or point mutations
Source: EMBO Mol Med. 2014 Feb 24;6(4):458–66. doi: 10.1002/emmm.201303672 (PMC3992073; doi:10.1002/emmm.201303672)
Supplement: Supplementary file 13 [file emmm0006-0458-sd13.pdf]

Supporting Table S2: DNA target sequence and amino acid sequences of the recognition helices of the four- or five-finger CD-specific ZFP constructs

Name	DNA target	Finger design					note*
		F1	F2	F3	F4	F5	
R8-1	ggAGGTAGGTGGTAgt	QSGALAR	RSDALAR	RSDNLSA	RSDHRIT		
R8-2	ggAGGTAGGTGGTAGTTtg	TSGSLSR	QSGSLTR	RSDALAR	RSDNLSA	RSDHRIT	
R8-3	ggAGGTAGGTGGTAGTTtg	TSGSLSR	QSGSLTR	RSDALSQ	RNDNRIT	RSDHLTQ	A
R8-4	ggAGGTAGGTGGTAGTTtg	TSGSLSR	QSGSLTR	RSDALSQ	RNDNRIT	RSDHLTQ	A
R8-5	ggAGGTAGGTGGTAGTTtg	TSGSLSR	QSGSLTR	RSDSLLR	RSDNLTT	RSDHLTQ	B
R8-6	ggAGGTAGGTGGTAGTTtg	TSGSLSR	QSGSLTR	RSDSLLR	RSDNLTT	RSDHLTQ	B
R8-7	ggGAGGTAgGTGGTAgt	QSGALAR	RSDALAR	QSGALAR	RSDNLTR		
R8-8	ggGAGGTAgGTGGTAGTTtg	TSGSLTR	QSGALAR	RSDALAR	QSGALAR	RSDNLTR	C
R8-9	ggGAGGTAgGTGGTAGTTtg	TSGSLTR	QSGALAR	RSDALAR	QSGALAR	RSDNLTR	C
R8-10	ggGAGGTAgGTGGTAGTTtg	TSGSLSR	QSGSLTR	RSDALAR	QSGALAR	RSDNLTR	D
R8-11	ggGAGGTAgGTGGTAGTTtg	TSGSLSR	QSGSLTR	RSDALAR	QSGALAR	RSDNLTR	D
R8-12	ggGAGGTAgGTGGTAGTTtg	TSGSLSR	QSGSLTR	TSGHLSR	QSGALAR	RSDNLTR	
R8-13	ggGAGGTAgGTGGTAGTTtg	TSGSLSR	QSGSLTR	TSGHLSR	QSGSLTR	RSDNLTR	
R13-1	caCCATTGGCAGCCTAGca	RSDNLST	DRSDLSR	QSGDLTR	RSDSLSA	QKATRIT	F
R13-2	caCCATTGGCAGCCTAGca	RSDNLST	DRSDLSR	QSGDLTR	RSDSLSA	QKATRIT	F

* Letters highlight design pairs that contain identical recognition helices, but differ at one or more linkers.

The ZFPs designed to bind in the vicinity of the 8470-8482 repeat are in red, whereas the ZFPs targeting DNA sequence in the vicinity of the 13447-13459 repeat are in blue. All presented ZFPs were designed and generated by Sangamo Biosciences Inc.
