# Supplementary material for: Mitochondrially targeted ZFNs for selective degradation of pathogenic mitochondrial genomes bearing large-scale deletions or point mutations
Source: EMBO Mol Med. 2014 Feb 24;6(4):458–66. doi: 10.1002/emmm.201303672 (PMC3992073; doi:10.1002/emmm.201303672)
Supplement: Supplementary file 14 [file emmm0006-0458-sd14.pdf]

Supporting Note S1: DNA sequences of mtZFN(+)/mtZFN(-) constructs

Legend:

Mitochondrial targeting sequence
Hemagglutinin
Flag
Nuclear export signal
Linkers
FokI(+) ELD
FokI(-) KKR
Residues changed in order to obtain the ELD (+) and KKR (-) versions of *Fok*I

mtZFN(+): MTS-HA-NES-ZFP-FokI(ELD):

ATGTTGGGGTTTGTGGGTCGGGTGGCCGCTGCTCCGGCCTCCGGGGCCTTGCGGAGACTCACCCCTTCAGCGTCGC
TGCCCCCAGCTCAGCTCTTACTGCGGGCCGCTCCGACGGCGGTCCATCCTGTCAGGGACTATGCGGCGCAATACCC
CTACGACGTGCCCGACTACGCCGTGGATGAAATGACCAAAAAGTTCGGCACGCTCACCATTCACGACACCGAAAAG
GCCGCCGAATTC

NARPd or R8-*n* zinc finger assemblies (Table S1)

GGATCCCAGCTGGTGAAGAGCGAGCTGGAGGAGAAGAAGTCCGAGCTGCGGCACAAGCTGAAGTACGTGCCCCACG
AGTACATCGAGCTGATCGAGATCGCCAGGAACAGCACCCAGGACCGCATCCTGGAGATGAAGGTGATGGAGTTCTT
CATGAAGGTGTACGGCTACAGGGGAAAGCACCTGGGCGGAAGCAGAAAGCCTGACGGCGCCATCTATACAGTGGGC
AGCCCCATCGATTACGGCGTGATCGTGGACACAAAGGCCTACAGCGGCGGCTACAATCTGCCTATCGGCCAGGCCG
ACGAGATGCAGAGATACGTG**A**AGGAGAACCAGACCCGGAATAAGCACATCAACCCCAACGAGTGGTGGAAGGTGTA
CCCTAGCAGCGTGACCGAGTTCAAGTTCCTGTTCGTGAGCGGCCACTTCAAGGGCAACTACAAGGCCCAGCTGACC
AGGCTGAACC**G**CA**AG**ACCAACTGCAATGGCGCCGTGCTGAGCGTGGAGGAGCTGCTGATCGGCGGCGAGATGATCA
AAGCCGGCACCCTGACACTGGAGGAGGTGCGGCGCAAGTTCAACAACGGCGAGATCAACTTCTGA **3'**

mtZFN(-): MTS-FLAG-NES-ZFP-FokI(KKR)

ATGTTGGGGTTTGTGGGTCGGGTGGCCGCTGCTCCGGCCTCCGGGGCCTTGCGGAGACTCACCCCTTCAGCGTCGC
TGCCCCCAGCTCAGCTCTTACTGCGGGCCGCTCCGACGGCGGTCCATCCTGTCAGGGACTATGCGGCGCAAGATTA
CAAGGACGACGATGACAAGGTGGATGAAATGACCAAAAAGTTCGGCACGCTCACCATTCACGACACCGAAAAGGCC
GCCGAATTC

COMPa or R13-*n* zinc finger assemblies (Table S1)

GGATCCCAGCTGGTGAAGAGCGAGCTGGAGGAGAAGAAGTCCGAGCTGCGGCACAAGCTGAAGTACGTGCCCCACG
AGTACATCGAGCTGATCGAGATCGCCAGGAACAGCACCCAGGACCGCATCCTGGAGATGAAGGTGATGGAGTTCTT
CATGAAGGTGTACGGCTACAGGGGAAAGCACCTGGGCGGAAGCAGAAAGCCTGACGGCGCCATCTATACAGTGGGC
AGCCCCATCGATTACGGCGTGATCGTGGACACAAAGGCCTACAGCGGCGGCTACAATCTGCCTATCGGCCAGGCCG
ACGAGATGGAGAGATACGTGGAGGAGAACCAGACCCGG**G**ATAAGCAC**C**T**G**AACCCCAACGAGTGGTGGAAGGTGTA
CCCTAGCAGCGTGACCGAGTTCAAGTTCCTGTTCGTGAGCGGCCACTTCAAGGGCAACTACAAGGCCCAGCTGACC
AGGCTGAACCACATCACCAACTGCAATGGCGCCGTGCTGAGCGTGGAGGAGCTGCTGATCGGCGGCGAGATGATCA
AAGCCGGCACCCTGACACTGGAGGAGGTGCGGCGCAAGTTCAACAACGGCGAGATCAACTTCTGA **3'**
