# Supplementary material for: Mitochondrially targeted ZFNs for selective degradation of pathogenic mitochondrial genomes bearing large-scale deletions or point mutations
Source: EMBO Mol Med. 2014 Feb 24;6(4):458–66. doi: 10.1002/emmm.201303672 (PMC3992073; doi:10.1002/emmm.201303672)
Supplement: Supplementary file 15 [file emmm0006-0458-sd15.pdf]

Supporting Note S2: DNA and protein sequence of CD-specific zinc fingers

R8-4

5’
ATGGCTGAGAGGCCCTTCCAGTGTCGAATCTGCATGCGTAACTTCAGTACCTCCGGCTCCCTGTCCCGCCACATCC
GCACCCACACAGGCGAGAAGCCTTTTGCCTGTGACATTTGTGGGAGGAAGTTTGCCCAGTCCGGCTCCCTGACCCG
CCATACCAAGATACACACGGGCGGACAACGGCCGTTCCAGTGTCGAATCTGCATGCGTAACTTCAGTCGTAGTGAC
GCCCTGAGCCAGCACATCCGCACCCACACAGGCGAGAAGCCTTTTGCCTGTGACATTTGTGGGAGGAAATTTGCCC
GGAACGACAACCGCATAACGCATACCAAGATACACACGGGCGAGAAGCCCTTCCAGTGTCGAATCTGCATGCGTAA
GTTTGCCCGCTCCGACCACCTGACCCAGCATACCAAGATACACCTGCGG **3’**

N:
MAERPFQCRICMRNFSTSGSLSRHIRTHTGEKPFACDICGRKFAQSGSLTRHTKIHTGGQRPFQCRICMRNFSRSD
ALSQHIRTHTGEKPFACDICGRKFARNDNRITHTKIHTGEKPFQCRICMRKFARSDHLTQHTKIHLR **:C**

R8-13

5’
ATGGCTGAGAGGCCCTTCCAGTGTCGAATCTGCATGCGTAACTTCAGTACCTCCGGCTCCCTGTCCCGCCACATCC
GCACCCACACAGGCGAGAAGCCTTTTGCCTGTGACATTTGTGGGAGGAAGTTTGCCCAGTCCGGCTCCCTGACCCG
CCATACCAAGATACACACGGGCGGAGGCGGAAGCCAACGGCCGTTCCAGTGTCGAATCTGCATGCGTAACTTCAGT
ACCTCCGGCCACCTGTCCCGCCACATCCGCACCCACACAGGCGAGAAGCCTTTTGCCTGTGACATTTGTGGGAGGA
AGTTTGCCCAGTCCGGCTCCCTGACCCGCCATACCAAGATACACACGGGATCTCAGAAGCCCTTCCAGTGTCGAAT
CTGCATGCGTAAGTTTGCCCGCTCCGACAACCTGACCCGCCATACCAAGATACACCTGCGG **3’**

N:
MAERPFQCRICMRNFSTSGSLSRHIRTHTGEKPFACDICGRKFAQSGSLTRHTKIHTGGGGSQRPFQCRICMRNFS
TSGHLSRHIRTHTGEKPFACDICGRKFAQSGSLTRHTKIHTGSQKPFQCRICMRKFARSDNLTRHTKIHLR **:C**

R13-1

5’
ATGGCTGAGAGGCCCTTCCAGTGTCGAATCTGCATGCGTAACTTCAGTCGCTCCGACAACCTGTCCACCCACATCC
GCACCCACACAGGCGAGAAGCCTTTTGCCTGTGACATTTGTGGGAGGAAGTTTGCCGACCGCTCCGACCTGTCCCG
CCATACCAAGATACACACGGGCGAGAAGCCCTTCCAGTGTCGAATCTGCATGCGTAAGTTTGCCCAGTCCGGCGAC
CTGACCCGCCATACCAAGATACACACGGGATCTCAGAAGCCCTTCCAGTGTCGAATCTGCATGCGTAACTTCAGTC
GCTCCGACTCCCTGTCCGCCCACATCCGCACCCACACAGGCGAGAAGCCTTTTGCCTGTGACATTTGTGGGAGGAA
GTTTGCCCAGAAGGCCACCCGCATCACCCATACCAAGATACACCTGCGG **3’**

N:
MAERPFQCRICMRNFSRSDNLSTHIRTHTGEKPFACDICGRKFADRSDLSRHTKIHTGEKPFQCRICMRKFAQSGD
LTRHTKIHTGSQKPFQCRICMRNFSRSDSLSAHIRTHTGEKPFACDICGRKFAQKATRITHTKIHLR **:C**

R13-2

5’
ATGGCTGAGAGGCCCTTCCAGTGTCGAATCTGCATGCGTAACTTCAGTCGCTCCGACAACCTGTCCACCCACATCC
GCACCCACACAGGCGAGAAGCCTTTTGCCTGTGACATTTGTGGGAGGAAGTTTGCCGACCGCTCCGACCTGTCCCG
CCATACCAAGATACACACGGGCGGACAACGGCCGTTCCAGTGTCGAATCTGCATGCGTAAGTTTGCCCAGTCCGGC
GACCTGACCCGCCATACCAAGATACACACGGGCGAGAAGCCCTTCCAGTGTCGAATCTGCATGCGTAACTTCAGTC
GCTCCGACTCCCTGTCCGCCCACATCCGCACCCACACAGGCGAGAAGCCTTTTGCCTGTGACATTTGTGGGAGGAA
GTTTGCCCAGAAGGCCACCCGCATCACCCATACCAAGATACACCTGCGGGGATCC **3’**

N:
MAERPFQCRICMRNFSRSDNLSTHIRTHTGEKPFACDICGRKFADRSDLSRHTKIHTGGQRPFQCRICMRKFAQSG
DLTRHTKIHTGEKPFQCRICMRNFSRSDSLSAHIRTHTGEKPFACDICGRKFAQKATRITHTKIHLRGS **:C**
