# Supplementary material for: Mitochondrially targeted ZFNs for selective degradation of pathogenic mitochondrial genomes bearing large-scale deletions or point mutations
Source: EMBO Mol Med. 2014 Feb 24;6(4):458–66. doi: 10.1002/emmm.201303672 (PMC3992073; doi:10.1002/emmm.201303672)
Supplement: Supplementary file 16 [file emmm0006-0458-sd16.pdf]

Supporting Note S3: DNA sequences of probes used in the *in vitro* testing of CD-specific ZFNs.

---8470-8482---

```
gtatggcccaccataattacccccatactccttacactattcctcatcacccaactaaaaatattaaa
cacaAACTACCACCTACCTccctcaccaaagcccataaaaataaaaaattataacaaaccctgagaac
caaaatgaacgaaaatctgttcgcttcattcattgcccccacaatcctaggcctacccgccgcagtac
tgatcattc
```

---13447-13459---

```
gccatactatttatgtgctccgggtccatcatccacaaccttaacaatgaacaagatattcgaaaaat
aggaggactactcaaaaccatacctctcacttcaacctccctcaCCATTGGCAGCCTAGcattagcag
gaatacctttcctcacaggtttctactccaaagaccacatcatcgaaaccgcaaacatatcatacaca
aacgcctgagccctatctattac
```

---8470-13447---

```
gtatggcccaccataattacccccatactccttacactattcctcatcacccaactaaaaatattaaa
cacaAACTACCACCTACCTccctcaCCATTGGCAGCCTAGcattagcaggaatacctttcctcacagg
tttctactccaaagaccacatcatcgaaaccgcaaacatatcatacacaaacgcctgagccctatcta
ttac
```

The binding sites for mtZFN monomers R8-*n*(+) and R13-*n*(-) on either side of the CD are in red and blue, respectively. Black boxes denote the mtDNA tandem repeats. Respective DNA fragments were PCR amplified from a total human DNA sample and the resulting product was cloned into the pCR4 plasmid using ZeroBlunt® TOPO® Cloning Kit (Life Technologies) according to the manufacturer's instructions. For the *in vitro* tests (**Fig. S1**) these pCR4 plasmids containing mtDNA fragments were digested with *Nco*I and end-labeled.
