# Supplementary material for: Mitochondrially targeted ZFNs for selective degradation of pathogenic mitochondrial genomes bearing large-scale deletions or point mutations
Source: EMBO Mol Med. 2014 Feb 24;6(4):458–66. doi: 10.1002/emmm.201303672 (PMC3992073; doi:10.1002/emmm.201303672)
Supplement: Supplementary file 17 [file emmm0006-0458-sd17.pdf]

SUPPORTING MATERIAL AND METHODS

Design and *in vitro* testing of ZFPs

Design and assembly of ZFPs specific to m.8993T>G mutation was carried out by Sangamo BioSciences and described previously (Minczuk *et al.*, 2008). A similar approach was taken in order to design and assemble ZFPs specific to CD, also carried out by Sangamo BioSciences. ZFPs were subjected to *in vitro* testing, as previously described (Minczuk, 2010) and presented in **Supporting Figure S4**.

Generation of mtZFN

ZFP sequences were cloned between 5' *Eco*RI and 3' *Bam*HI sites in a pcDNA3.1(-) (Life Technologies) backbone containing upstream MTS-NES-[HA/FLAG]-linker and downstream linker-FokI (+/-). The sequences of the mtZFN ORFs are given in Constructs containing R13-*n*(-) or COMPa(-) mtZFNs were then digested with PmeI and blunt-end cloned into pTracer CMV/Bsd (Life Technologies). All cloning procedures were carried out using standard molecular biology techniques and reagents. The sequences are given in **Supporting Notes S1** and **S2**.

Two-dimensional agarose gel electrophoresis

For two-dimensional gels, DNA was extracted by sequential phenol-chloroform extraction according to established protocols (Reyes *et al.*, 2007) from mitochondria isolated by methods described previously (see Cell fractionation). 7µg of DNA was restricted according to manufacturer's instructions (New England Biolabs) and separated on agarose gels according to protocols described in detail elsewhere (Reyes *et al.*, 2007). Gels were then blotted and hybridised as above, followed by washing with 1 × SSC three times for 20 min, then 1 × SSC with 0.1%

SDS three times for 20 min. Primer sequences used to produce the probes are given in main text.

Extracellular flux analysis

Clones obtained from transfections were seeded at 3 x 10^4 cells/well in 200ml growth medium in XF24-well cell culture microplates (Seahorse Bioscience) and incubated at 37 °C in 5 % CO_2 for 24 h. One hour before the assay growth medium was removed and replaced with assay medium (low buffered DMEM, 10 mM L-glutamine, 1 mM sodium pyruvate, 2 mM glucose), with one wash of assay medium, and left to stabilise in a 37 °C non-CO_2 incubator. Analysis was performed in quadruplicate using a XF24 Extracellular Flux Analyzer (Seahorse Bioscience). The wells were sequentially injected with 20mM 2-deoxyglucose (2-DG) to inhibit glycolysis, 100 nM oligomycin to inhibit ATP-synthase, 500–1000 nM carbonylcyanide-4-trifluorometho-xyphenylhydrazone (FCCP) to uncouple the respiratory chain and 200 nM rotenone to inhibit complex I. Oxygen consumption rate (OCR) was measured every 5 min. Test compounds: 2-DG, oligomycin, FCCP and rotenone were all obtained from Sigma.
