# Supplementary material for: Mitochondrially targeted ZFNs for selective degradation of pathogenic mitochondrial genomes bearing large-scale deletions or point mutations
Source: EMBO Mol Med. 2014 Feb 24;6(4):458–66. doi: 10.1002/emmm.201303672 (PMC3992073; doi:10.1002/emmm.201303672)
Supplement: Supplementary file 18 [file emmm0006-0458-sd18.pdf]

SUPPORTING REFERENCES

Minczuk M Engineered zinc finger proteins for manipulation of the human mitochondrial genome. Methods Mol Biol 2010;649:257-270.

Minczuk M, Kolasinska-Zwierz P, Murphy MP, and Papworth MA Construction and testing of engineered zinc-finger proteins for sequence-specific modification of mtDNA. Nat Protoc 2010;5:342-356.

Minczuk M, Papworth MA, Miller JC, Murphy MP, and Klug A Development of a single-chain, quasi-dimeric zinc-finger nuclease for the selective degradation of mutated human mitochondrial DNA. Nucleic Acids Res 2008;36:3926-3938.

Reyes A, Yasukawa T, and Holt IJ Analysis of replicating mitochondrial DNA by two-dimensional agarose gel electrophoresis. Methods Mol Biol 2007;372:219-232.
